# Reverse Engineering Time Discrete Finite Dynamical Systems: A Feasible Undertaking?

**DOI:** 10.1371/journal.pone.0004939

**Published:** 2009-03-19

**Authors:** Edgar Delgado-Eckert

**Affiliations:** 1 Centre for Mathematical Sciences, Technische Universität München, Garching, Germany; 2 Pathology Department, Tufts University, Boston, Massachusetts, United States of America; IBM Thomas J. Watson Research Center, United States of America

## Abstract

With the advent of high-throughput profiling methods, interest in reverse engineering the structure and dynamics of biochemical networks is high. Recently an algorithm for reverse engineering of biochemical networks was developed by Laubenbacher and Stigler. It is a top-down approach using time discrete dynamical systems. One of its key steps includes the choice of a term order, a technicality imposed by the use of Gröbner-bases calculations. The aim of this paper is to identify minimal requirements on data sets to be used with this algorithm and to characterize optimal data sets. We found minimal requirements on a data set based on how many terms the functions to be reverse engineered display. Furthermore, we identified optimal data sets, which we characterized using a geometric property called “general position”. Moreover, we developed a constructive method to generate optimal data sets, provided a codimensional condition is fulfilled. In addition, we present a generalization of their algorithm that does not depend on the choice of a term order. For this method we derived a formula for the probability of finding the correct model, provided the data set used is optimal. We analyzed the asymptotic behavior of the probability formula for a growing number of variables n (i.e. interacting chemicals). Unfortunately, this formula converges to zero as fast as 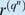, where 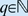 and 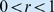. Therefore, even if an optimal data set is used and the restrictions in using term orders are overcome, the reverse engineering problem remains unfeasible, unless prodigious amounts of data are available. Such large data sets are experimentally impossible to generate with today's technologies.

## Introduction

Since the development of multiple and simultaneous measurement techniques such as microarray technologies, reverse engineering of biochemical and, in particular, gene regulatory networks has become a more important problem in systems biology. One well-known reverse engineering approach is that of top-down methods, which try to infer network properties based on the observed global input-output-response. The observed input-output-response is usually only partially described by available experimental data.

Depending on the type of mathematical model used to describe a biochemical process, a variety of top-down reverse engineering algorithms have been proposed [1], [2], [3]. See also [4] for probabilistic approaches. Each modeling paradigm presents different requirements relative to quality and amount of the experimental data needed. Moreover, for each type of model, a suitable mathematical framework has to be developed in order to study the performance and limitations of reverse engineering methods. For any given modeling paradigm and reverse engineering method it is important to answer the following questions:

1. What are the minimal requirements on data sets?
2. Can data sets be characterized in such a way that “optimal” data sets can be identified? (Optimality meaning that the algorithm performs better using such a data set compared to its performance using other data sets.)

The second question is related to the *design of experiments* and optimality is characterized in terms of *quantity and quality* of the data sets. Some algebraic approaches dealing with issues related to the design of statistical experiments have yielded problems that are algebraically similar to the above questions (put in the context of this paper). In particular, in the relatively new field of *algebraic statistics*, Gröbner-bases theory (see below) has been used to address similar issues. Some of the findings on this topic and also some of the limitations attached to the use of term orders (to be defined below) can be found in [5] and [6].

The authors of [7] developed a top-down reverse engineering algorithm for the modeling paradigm of time discrete finite dynamical systems. Herein, we will refer to it as the LS-algorithm. They apply their method to biochemical networks by modeling the network as a time discrete finite dynamical system, which is obtained by discretizing the concentration levels of the interacting chemicals to elements of a finite field. One of the key steps of the LS-algorithm includes the choice of a term order, a technicality imposed by the use of Gröbner-bases calculations (see, for instance, [8]). The modeling paradigm of time discrete finite dynamical systems generalizes the Boolean approach [9] (where the field only contains the elements 0 and 1). Moreover, it is a special case of the paradigm described in [10], in which asynchronous updating of the state variables is allowed.

Some aspects of the performance of the LS-algorithm were studied by the author of [11] in a probabilistic framework. Specifically, the author of [11] explores a somewhat different question, namely, how many *randomly generated* data points are needed on average before the LS-algorithm finds the correct model (we will give a precise definition of “correct model”). To this end, the author of [11] assumes that information about the actual number of interactions (or an upper bound for this number) in the biochemical network is available. Furthermore, two particular classes of term orders are considered in the analysis. Many of the bounds derived by the author of [11] for the necessary length of a data set to provide enough information, are bounded below by 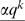 or 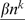, where *n* is the total number of species, 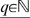, 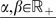 are positive real constants and *k* is an upper bound for the number of species affecting the entity whose function is to be reverse engineered. As a consequence, even in the case of a relatively small biochemical network involving only *n = 30* entities, to successfully reverse engineer a function depending on only *k = 5* variables would require (according to the results presented in [11]) about 30^5^ = 24.3 million random experiments. We consider this outcome of the analysis by the author of [11] rather discouraging from an experimental point of view. It is also an open question to what extent it is realistic to assume that biological or biochemical experiments can be massively performed in a randomized manner.

In this paper we investigate the two questions stated above in the particular case of the LS-algorithm. For this purpose, we developed a mathematical framework that allows us to study the LS-algorithm in depth. Having expressed the steps of the LS-algorithm in our framework, we were able to provide concrete answers to both questions: First, we found minimal requirements on a data set based on how many terms the functions to be reverse engineered display. Second, we identified optimal data sets, which we characterize using a geometric property called “general position”. Moreover, we developed a constructive method to generate optimal data sets, provided a codimensional condition is fulfilled.

In addition, we present a generalization of the LS-algorithm that does not depend on the choice of a term order. We call this generalization the *term-order-free reverse engineering method*. For this method we derive a formula for the probability of finding the correct model, provided the data set used satisfies an optimality criterion. Furthermore, we analyze the asymptotic behavior of the probability formula for a growing number of variables n (i.e. interacting chemicals). Unfortunately, this formula converges to zero as fast as 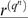, where 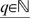 and 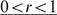. Consequently, we conclude that even if an optimal data set is used and the restrictions imposed by the use of term orders are overcome, the reverse engineering problem remains unfeasible, unless experimentally impracticable amounts of data are available. This result discouraged us from elaborating on the algorithmic aspects of the term-order-free reverse engineering method.

In [12], [13] and [14] the weaker problem of finding the causal (*static*) relationships between the variables in the network (as opposed to reverse engineering the *dynamical* properties of the network, which automatically provides the dependencies between the variables) has been studied in the context of the LS-algorithm. However, neither of the two questions stated above was addressed in those publications. In [14], the authors make use of the Gröbner fan to take into account all possible term orders and produce a consensus graph representing the most likely dependency relations among the nodes in the network. While this approach is helpful for finding the causal relationships between the variables, it still does not circumvent the issues related to the use of term orders when it comes to the more challenging task of reverse engineering the dynamical properties of the network. This is because the Gröbner fan only comprises term orders.

In contrast to [11], we focus here on providing possible criteria for the design of specific experiments instead of assuming that the data sets are generated randomly. Moreover, we do not necessarily assume that information about the actual number of interactions in the biochemical network is available.

The organization of this article is the following:

The Methods Section is devoted to the mathematical background: We briefly describe the LS-algorithm and provide a mathematical framework to study it. Moreover, we introduce the term-order-free reverse engineering method. The Results Section presents rigorous results and some of their consequences. In the Discussion Section we summarize our main results, discuss their consequences and provide further conclusions.

To fully understand the technical details of our analysis, very basic knowledge in linear algebra and algebra of multivariate polynomials is required. We have included a series of endnotes to provide some guidance. Nevertheless, we refer the interested reader to [15] and [8].

## Methods

### Mathematical background

#### A short description of the LS-algorithm

We encourage the interested reader to read the original work [7], where the LS-algorithm is introduced. We also refer to 2.1 in [11] for another mathematical description of the LS-algorithm. However, for the sake of completeness, in this subsection we describe the LS-algorithm and its basic mathematical properties.

In the modeling paradigm described in [7], a biological or biochemical system is described by *n* time varying quantities *s_1_(t)*,*…*,*s_n_(t)*, which represent the state of the system at the point in time *t*. The evolution of the system is observed by taking *m* consecutive measurements of each of the interacting quantities. This yields one time series
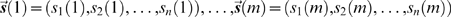
Such series of consecutive measurements are repeated *l* times starting from different initial conditions, where the length 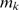 of the series may vary. At the end of this experimental procedure, several time series are obtained:
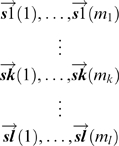
Each point in a time series is a vector or *n*-tuple in 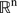, where 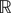 is the set of all real numbers. Time series are then discretized using a discretization algorithm (see, for instance, [16]) that can be expressed as a map
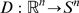
(1)where the set *S* is a finite field^1^ of cardinality 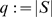 (the cardinality of the field used is determined during the discretization process). The discretized time series can be written as
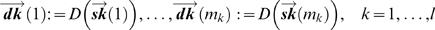
One fundamental assumption made in their paper is that the evolution in time of the discretized vectors obeys a simple rule, namely, that there is a function
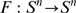
such that
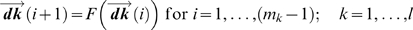
(2)The authors of [7] call *F* the transition function of the system. One key ingredient in the LS-algorithm is the fact that the set *S* is endowed with the algebraic structure of a finite field. Under this assumption, the rule (2) reduces to a polynomial interpolation problem in each component, i.e. for each 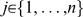


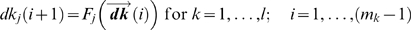
(3)The information provided by the equations (3) usually underdetermines the function 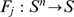, unless for all possible vectors 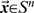, the values 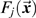 are established by (3). Indeed, any non-zero polynomial function that vanishes on the data inputs
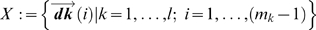
could be added to a function satisfying the conditions (3) and yield a different function that also satisfies (3). Among all those possible solutions, the LS-algorithm chooses an interpolating polynomial function 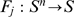 that does not contain any terms vanishing on the set *X*. Unfortunately, the LS-algorithm works within an algebraic framework that depends on the choice of a so called term order. For every different term order, the output of the algorithm might be a different one. In addition, term orders impose some quite arbitrary conditions on the set of possible candidates for the output of the LS-algorithm. Furthermore, there is no clear criterion when it comes to actually choosing a term order. In the next subsection we will provide the definition of term order as well as a geometric framework in which the algebraic steps of the LS-algorithm can be visualized and better understood. In Section 1 of the Appendix S1, we provide a concrete example in which the output of the algorithm is clearly presented.

For the sake of completeness, we summarize here the technical steps of the LS-algorithm: To generate its output, the algorithm first takes as input the discretized time series and generates functions 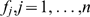 that satisfy (3) for each 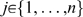 correspondingly. Secondly, it takes a monomial order 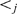 as input and generates the normal form of 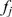 with respect to the vanishing ideal 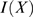 and the given order 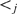. For every 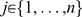, this normal form is the output 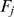 of the algorithm.

#### A mathematical framework to study the reverse engineering problem

The mathematical framework presented here is based on a general algebraic result presented by the author in Section 4 of the Appendix S1. This result is known among algebraists, however, to the author's best knowledge, it has never been formulated within the context considered herein. This framework will allow us to study the LS-algorithm as well as a generalized algorithm of it that is independent on the choice of term orders. Furthermore, within this framework, we will be able to provide answers to the two questions stated in the Introduction, (see the Results section below). In this sense, this subsection “sets the stage” for our investigations. We use several well established linear algebraic results to construct the framework within which our investigations can be carried out.

We start with the original problem: Given a time-discrete dynamical system over a finite field *S* in *n* variables
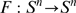
and a data set 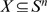 generated by iterating the function *F* starting at one or more initial values, what are the chances of reconstructing the function *F* if the LS-algorithm or a similar algorithm is applied using *X* as input time series?

From an experimental point of view the following question arises: What is the function *F* in an experimental setting? Contrary to the situation when models with an infinite number of possible states are reverse engineered (see 1.2 in [17]), there is a finite number of experiments that could, at least theoretically, be performed to completely characterize the system studied. In this sense, even in an experimental setting, there is an underlying function *F*. The components of this function is what the author of [11] called 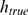.

Since the algorithms studied here generate an output model 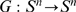 by calculating every single coordinate function 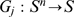 separately, we will focus on the reconstruction of a single coordinate function 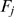 which we will simply call *f*. We will use the notation 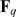 for a finite field of cardinality 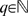. In what follows, we briefly review the main definitions and results stated and proved in Section 4 of the Appendix S1:

We denote the 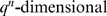 vector space of functions 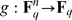 with 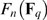. A basis for 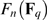 is given by all the monomial functions
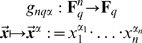
where the exponents 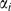 are non-negative integers satisfying 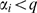. The basis of all those monomial functions is denoted with 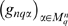, where
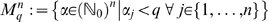
We call those monomial functions *fundamental monomial functions*. This fact is basically telling us that all functions 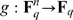 are polynomial functions of bounded degree^2^.

When dealing with polynomial interpolation problems, it is convenient to establish the relationship between a polynomial function 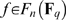 and the value it takes on a given point 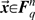 or set of points 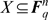. A technique commonly used in algebra is to define an evaluation mapping that assigns to each polynomial function 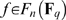 the list of the values it takes on each point 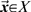 of a given set of different points 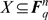. Just to make sure this mapping is unique, we order this list of evaluations according to a fixed but arbitrary order. This is equivalent to ordering the set 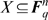 in the first place (see endnote 4 in the next page). Summarizing, consider a given finite field 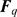, natural numbers 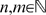 with 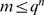 and an (ordered) tuple
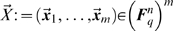
of *m* **different** points with entries in the field 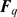. Then we can define the mapping
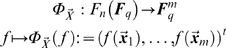
(where *t* denotes transpose). It can be shown (see Theorem 21 in Section 4 of the Appendix S1), that this mapping is a surjective linear operator^3^. We call this mapping the evaluation epimorphism of the tuple 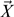.

**Figure 1:**
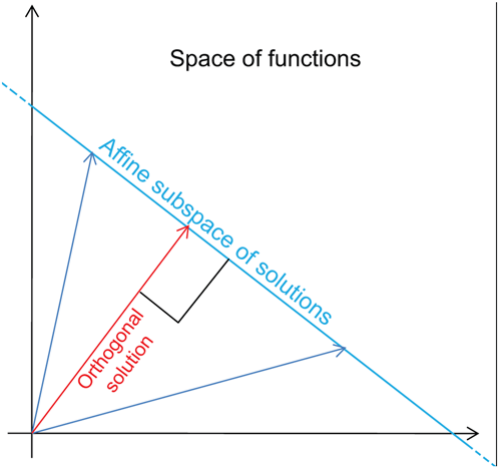
The set of all solutions to the polynomial interpolation problem is an affine subspace. A two-dimensional representation of the space of functions 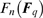. Within this space, a one-dimensional representation of the affine subspace of solutions of 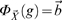. Three particular solutions are depicted; one (red) is the orthogonal solution.

For a given set 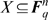 of data points and a given vector 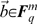, the interpolation problem of finding a function 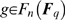 with the property
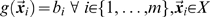
can be expressed using the evaluation epimorphism as^4^ follows: Find a function 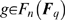 with the property
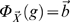
(4)Since a basis of 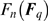 is given by the fundamental monomial functions 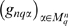 , the matrix^5^

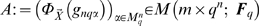
representing the evaluation epimorphism 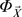 of the tuple 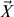 with respect to the basis 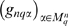 of 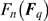 and the canonical basis of 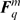 has always the full rank 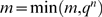. That also means, that the dimension of the 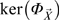 is^6^

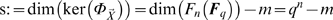
(5)In the case 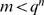 where *m* is strictly smaller than 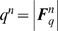 we have 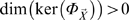 and the solution of the interpolation problem is not unique. There are exactly 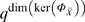 different solutions which constitute an affine subspace of 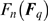 (see Fig. 1). Only in the case 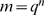, that means, when for all elements of 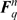 the corresponding interpolation values are given, the solution is unique. Experimental data are typically sparse and therefore *underdetermine* the problem. If the problem is underdetermined and no additional information about properties of the possible solutions is given, any algorithm attempting to solve the problem has to provide a *selection criterion* to pick a solution among the affine space of possible solutions. If we visualize the affine subspace of solutions of (4) in the space 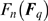 (see Fig. 1), among all possible solutions, the one that geometrically seems to capture the essential part of a solution is the one perpendicular to the affine subspace. This solution does not contain any components pointing in the direction of the subspace, which, at least geometrically, seem redundant.

Interestingly, this simple geometric idea comprises the algebraic selection step in the LS-algorithm and at the same time generalizes the pool of possible candidates to be selected. Of course we need to formalize this approach algebraically. The standard tool in this context is called *orthogonality*. For orthogonality to apply, a generalized inner product (see [15]) has to be defined on the space 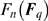. We finish this subsection reviewing these concepts (cf. Appendix S1).

The space 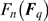 is endowed with a symmetric bilinear form^7^

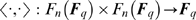
i.e. a generalized inner product. Two functions 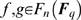 are called *orthogonal* if it holds 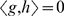. A family of functions 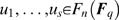 is called *orthonormal* if it holds^8^ 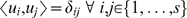.

For a given set 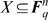 of data points, consider the evaluation epimorphism 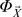 of the tuple 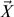 and its kernel 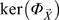. Now, let 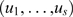 be a basis of 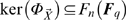. By the basis extension theorem (see [15]), we can extend the basis 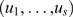 to a basis 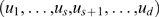 of the whole space 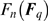, where 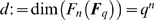. (There are many possible ways this extension can be performed. See more details below). As in Example 5 of Subsection 4.2.1 in the Appendix S1, we can construct a generalized inner product on 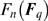 by setting^9^

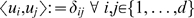
(6)The *orthogonal solution* of (4) is the solution 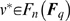 that is orthogonal to 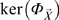, i.e. it holds 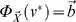 and for an arbitrary basis 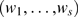 of 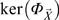 the following orthogonality conditions hold
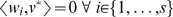



The way we extend the basis 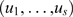 of 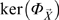 to a basis 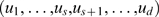 of the whole space 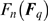 determines crucially the generalized inner product we get by setting (6). Consequently, the orthogonal solution of (4) may vary according to the extension 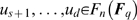 chosen. In the Appendix S1, a systematic way to extend the basis 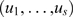 to a basis for the whole space is introduced. With the basis obtained, the process of defining a generalized inner product according to (6) is called the *standard orthonormalization*. This is because the basis 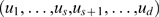 is orthonormal with respect to the generalized inner product defined by (6).

A basis is by definition an ordered set. The basis of fundamental monomial functions 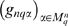 is an ordered set arranged according to a fixed order relation defined on the set 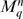. The most general partial order^10^ “<” that still allows for a unique arrangement of a finite set of elements is a *linear order*. A linear order < on the set 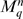 is a partial order such that, for every pair of elements 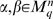, exactly one of the three statements
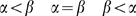
holds. Gröbner bases calculations, which are part of the LS-algorithm, require a specific way to order the terms in a polynomial. Such order relations are called *term orders*. One of the key requirements for a term order is that it must be consistent with the algebraic operations performed with polynomials. In particular, the term order relation must be preserved after multiplication with an arbitrary term. Additionally, it has to be possible to always determine which is the smallest element among a set of arbitrary terms. Since every term in a polynomial in *n* indeterminates is uniquely determined by the exponents appearing in it, the order relation can as well be defined on the set 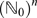 of tuples of non negative integer exponents. As stated above, in the context of polynomial functions in *n* variables over the finite field 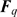, the degrees are bounded above and therefore we only need to consider the order relation on the set 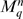. Let us consider a simple example in the case *n = 1* and *p = 5*. The terms 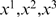 could be ordered according to a linear order > as
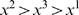
This order cannot be a term order. If it was a term order, then we could multiply both sides of the expression 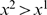 (which holds by transitivity) by 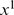 to obtain 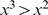. This result contradicts the order relation established above.

Essentially, the standard orthonormalization consists of two steps

1. Gaussian elimination (see [15]) on the coordinate vectors with respect to the basis 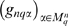 of a basis of 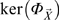.
2. Extension of the basis according to the columns in which no pivot element could be found during the Gaussian elimination in step 1).

The precise definition of the standard orthonormalization procedure together with an example is provided in Subsection 4.4 of the Appendix S1. The standard orthonormalization process depends on the way the elements of the basis 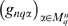 of fundamental monomial functions are ordered. If they are ordered according to a term order, the calculation of the orthogonal solution of (4)^11^ yields precisely the same result as the LS-algorithm. If more general linear orders are allowed, a more general algorithm emerges that is not restricted to the use of term orders. This algorithm can be seen as a generalization of the LS-algorithm. We call it the *term-order-free reverse engineering method*. In the next subsection we meticulously present the steps of the term-order-free reverse engineering method. It is pertinent to emphasize that although the term-order-free reverse engineering method generates the same solution as the LS-algorithm (provided we use a term order to order the elements of the basis 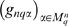), the two algorithms differ significantly in their steps. The steps of the LS-algorithm are defined in an algebraic framework that makes use of Gröbner bases calculations. This algebraic framework imposes restrictions on the type of order relations that can be used. Our method is defined in a geometric and linear algebraic framework that is not subjected to those restrictions. As a consequence, our method represents a generalization of the LS-algorithm in terms of the “spectrum” of solutions it can produce for a given input data set. Moreover, the fact that our method is capable of reproducing the input-output behavior of the LS-algorithm, allows us to study this behavior of the LS-algorithm within our, in our opinion, more tractable framework. In Section 1 of the Appendix S1 we present an illustrative example in which every step of the term-order-free reverse engineering method is carried out explicitly.

As we will show in the Results section, the monomial functions 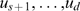 generated by the standard orthonormalization procedure to extend the basis 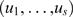 of 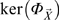 to a basis 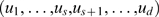 of the whole space 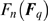 constitute the *pool of candidate monomials* for the construction of the orthogonal solution. In other words, the orthogonal solution is a linear combination of the 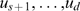.

The use of term orders is a requirement imposed by the algebraic approach used in the LS-algorithm. However, it arbitrarily restricts the ways the basis 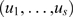 of 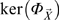 can be extended to a basis 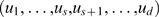 by virtue of the standard orthonormalization procedure. For instance, the constant function
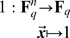
is always part of the extension 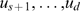 when term orders are used. This follows from the fact that for any term order > the property
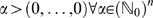
always holds (see Chapter 2, §4, Corollary 6 in [8]). Furthermore, if an optimal data set (to be defined below) is used, some high degree monomials will never be among the candidates . Thus, a function *f* displaying such high degree terms could never be reverse engineered by the LS-algorithm, if fed with an optimal data set.

It will also become apparent in the Results section, that the use of term orders makes it difficult to analyze the performance of the LS-algorithm.

As a consequence, we tried to circumvent the issues related to the use of term orders by proposing the term order free reverse engineering method, a generalization of the LS-algorithm that does not depend on the choice of a term order.

### The term-order-free reverse engineering method

Let 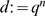. The input of the term-order-free reverse engineering algorithm is a set 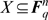 containing 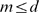 different data points, a list of *m* interpolation conditions
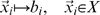
and a linear order < for the elements of the basis 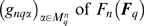, (i.e., the elements of the basis are ordered decreasingly according to < ). The steps of the algorithm are as follows

1. Calculate the entries of the matrix 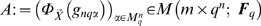 representing the evaluation epimorphism 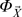 of the tuple 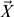 with respect to the basis 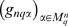 of 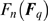 and the canonical basis of 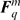.
2. Calculate the coordinate vectors (with respect to the basis 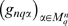) 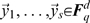 of a basis of 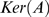.
3. Extend the basis 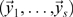 to a basis 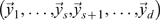 of 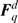 using the standard orthonormalization procedure (See Subsection 4.4 of the Appendix S1).
4. Define a generalized inner product 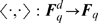 by setting
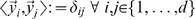
and calculate the entries of the matrix
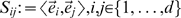
where 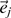 is the *j*th canonical unit vector of 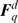.
5. The coordinate vector with respect to the basis 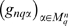 of the output function (the orthogonal solution) is obtained by solving the following system of inhomogeneous linear equations
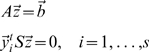

The steps described above represent an intelligible description of the algorithm and are not optimized for an actual computational implementation. In Section 1 of the Appendix S1 we present an illustrative example in which every step of the method is carried out explicitly.

Essentially, the steps of the term-order-free reverse engineering comprise standard matrix and linear algebra calculations. However, the size or dimension of the matrices involved depends exponentially on the number *n* of variables and linearly on the number *m* of data points, as the reader can verify based on the dimensions of the matrices involved in the algorithm. The complexity of basic linear algebraic calculations such as Gaussian elimination and back substitution are well known, see, for instance, [18]. With that in mind, we can briefly assess the complexity of our method: In step 1, 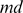 matrix entries need to be calculated as the evaluation of the fundamental monomial functions on the data points. In step 2, a basis of the nullspace of *A* is calculated. The number of data points *m* should be expressed as a proportion of the size of the entire space 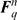, thus, we write 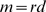 with a suitable factor 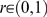. The basis of the nullspace is calculated using Gaussian elimination, which, neglecting the lower order terms in *d*, requires 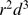 operations, and back substitution, which, given that 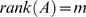, is 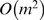. The standard orthonormalization procedure in step 3 is also accomplished via Gaussian elimination on an 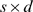 matrix. Due to 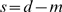, we have 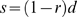, therefore, step 3 requires about 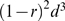 operations. The calculation of the matrix *S* in step 4 requires the inversion of a matrix, whose columns are precisely the extended basis coordinate vectors 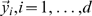. This inverted matrix is then multiplied by its transpose. The resulting product is the matrix *S* (see Example 1 in the Appendix S1 for more details). Thus, step 4 requires 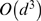 operations. Finding the solution of the *d*-dimensional system of linear equations in step 5 requires again 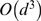 operations.

According to [7], the LS-algorithm is quadratic in the number *n* of variables and exponential in the number *m* of data points.

The exponential complexity of this type of algorithms should not be surprising, for it is an inherent property of even weaker reverse engineering problems (see [19]). Therefore, a computational implementation of these algorithms should take advantage of parallelization techniques and eventually of quantum computing.

The ill-conditioned dependency of the reverse engineering problem on the amount of input data needed (see Results section below) discouraged us from further elaborating on potential algorithmic improvements (for instance, using an extension of the Buchberger-Möller algorithm, [20], to calculate 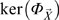) for the term-order-free reverse engineering method.

## Results

### Basic definitions, well known facts and some notation

For what follows recall that 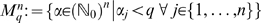. Let *K* be an arbitrary finite field, 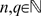 natural numbers and 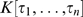 the polynomial ring in *n* indeterminates over *K*. It is a well known fact (see, for instance, [21], [22] and [8]) that the set of all polynomials of the form
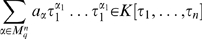
with coefficients 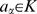 is a vector space over *K*. We denote this set with 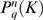.It is not surprising (see, for instance, [21], [22] and [8]) that the vector space 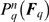.is isomorphic to the space 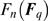 of functions in *n* variables defined on 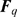. We denote the one-to-one mapping
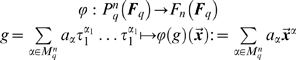
(6’)between these spaces with 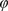.

In order to explore the LS-algorithm, we need the notion of “Ideal”, which is very common in commutative algebra and algebraic geometry (see, for instance, [8]):

**Definition 1** *Let K be a field*, 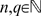 *natural numbers and* 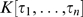 *the polynomial ring in n indeterminates over K. Furthermore, let* 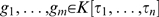 *be polynomials. The set*

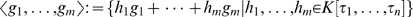

*is called the* ideal generated by 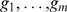 .

For a given set 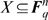 of data points and a given vector 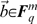, consider the evaluation epimorphism 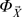 of the tuple 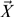 and its kernel 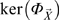. In addition, consider a fixed linear ordering *<* by which the elements of the basis 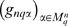 are ordered. In what follows, 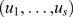 will be a basis of 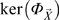. This basis will be extended to a basis 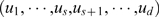 of the whole space 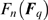, according to the standard orthonormalization procedure. The orthogonal solution of 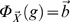 will be defined in terms of the generalized inner product defined by (6).

### Conditions on the data set

In this subsection, by virtue of the mathematical framework developed in the Methods section, we will address the following two problems regarding the LS-algorithm and its generalization, the term-order-free reverse engineering method:

**Problem 2** *Given a function* 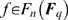, *what are the minimal requirements on a set* 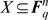, *such that the LS-algorithm reverse engineers f based on the knowledge of the values that it takes on every point in the set* 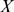 *?*

**Problem 3** *Are there sets* 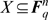 *that make the LS-algorithm more likely to succeed in reverse engineering a function* 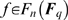 *based only on the knowledge of the values that it takes on every point in the set* 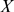 *?*

It is pertinent to emphasize that, contrary to the scenario studied in [11], we do not necessarily assume that information about the number of variables actually affecting *f* is available. We will give further comments on this issue at the end of the Discussion.

**Definition 4** *Let* 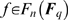, *be a polynomial function. The subset of* 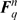 *containing all values on which the polynomial function f vanishes is denoted by* 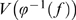, *where* 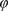 *is the mapping defined in* *equation (6’)* *(see previous subsection)*.

The following result tells us that if we are using the LS-algorithm to reverse engineer a nonzero function we necessarily have to use a data set 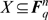 containing points where the function does not vanish.

**Theorem 5** *Let* 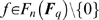 *be a nonzero polynomial function. Furthermore let*

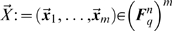

*be a tuple of m* ***different*** *n-tuples with entries in the field* 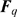, 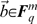 *the vector defined by*

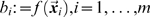

*and* 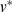 *the orthogonal solution of* 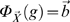. *Then if* 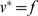, *it follows^12^*

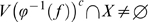



**Proof:** If 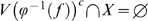, then by definition of 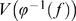, the vector 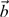 would be equal to the zero vector 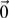. From Corollary 10 in Subsection 4.2.2 of the Appendix S1, we know that the orthogonal solution 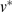 of 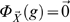 is the zero function, thus 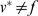.▪

**Theorem 6** *Let* 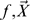 *and* 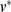 *be as in the previous theorem*. *In addition, assume* 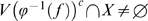. *Then it holds*

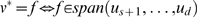



**Proof:** The claim follows directly from the definition of orthogonal solution and its uniqueness (see Section 4 of the Appendix S1 for more details).

**Remark 7** *From the necessary and sufficient condition*

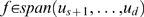
(7)
*it becomes apparent, that if the function f is a linear combination of more than* 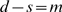 *fundamental monomial functions, f can not be found as an orthogonal solution* 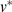 *of* 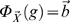 *(where* 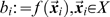 *). In particular, if f is a linear combination containing all d fundamental monomial functions in* 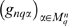, *no proper subset* 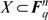 *of* 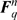 *will allow us to find f as orthogonal solution of* 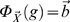.

**Remark 8** *From the condition (7) it follows that in order to reverse engineer a monomial function appearing in f using the term-order-free reverse engineering method or the LS-algorithm, it is necessary that the monomial function is linearly independent of the basis vectors* 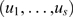 *of* 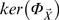. *For this reason, the set X should be chosen in such a way that no fundamental monomial function* 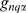 *is linearly dependent on the basis vectors* 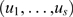 *of* 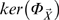. *Otherwise, some of the terms appearing in f might vanish on the set X and would not be detectable by any reverse engineering method, (as stated in* [*7*] *). This problem introduces a more general question about the existence of vector subspaces in “general position”:*

**Definition 9** *Let W be a finite dimensional vector space over a finite field* 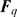 *with dim(W) = d>0. Furthermore, let* 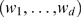 *be a fixed basis of W and* 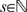 *a natural number with s<d. A vector subspace U⊂W with dim(U) = s is said to be in* general position *with respect to the basis* 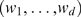, *if for any basis* 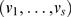 *of U and any injective mapping*

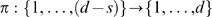

*the vectors*

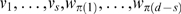

*are linearly independent*

**Remark 10** *Note that if U is in general position with respect to the basis* 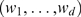, *then, for any permutation* 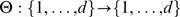 *of the elements of the basis* 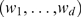, *the general position of U remains unchanged. In other words, U is in general position with respect to the permuted basis* 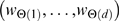.

**Figure 2:**
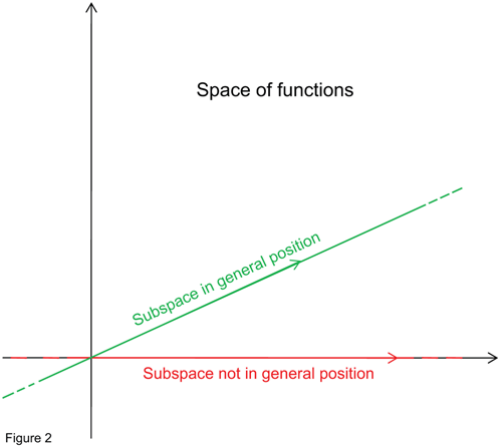
The notion of general position. A two-dimensional representation of the space of functions 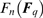. Within this space, two one-dimensional subspaces are depicted. One subspace (green) is in general position, while the other one (red) is not.

Figure 2 shows two one-dimensional subspaces. The red subspace is not in general position since its basis cannot be extended to a basis of the entire space (2 dimensions) by adjoining the first canonical unit vector (horizontal black arrow) to it.

It can be shown, that if the cardinality *q* of the finite field 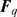 is sufficiently large, proper subspaces in general position of any positive dimension always exist. The proof is provided in Section 3 of the Appendix S1.

Now assume that 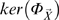 is in general position with respect to the basis 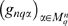 of 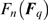. By the basis extension theorem and due to the general position of 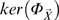 , we can extend the basis 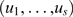 *of* 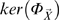 to a basis
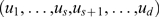
of the whole space 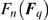, where 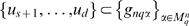 can be any subset of 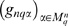 with *d-s* elements. Now we can construct a generalized inner product on 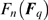 by setting (6). The advantage in this situation is that there is no bias imposed by the data on the monomial functions that can be used to extend the basis 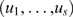 to a basis of 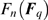. In addition, having this degree of freedom, it is possible to calculate the exact probability of success of the method. This probability depends of course on the number of fundamental monomial functions actually contained in *f*. We will give an explicit probability formula in the next Subsection. For our further analysis we need the following well known result (for a proof, see, for instance, [8]):

**Lemma and Definition 11** *Let* 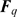 *be a finite field and* 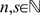 *natural numbers with* 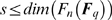. *Furthermore, let* 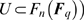 *be an s-dimensional subspace. Then the set*

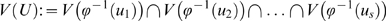

*where* 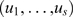 *is any basis of U, is independent on the choice of basis and it is called the* variety of the subspace *U*.

Now the following question arises: How should the set *X* be chosen in order to have 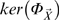 in general position with respect to the basis 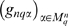
? A possible approach to this issue is the following: For a given natural number 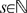 with 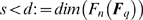, start from a basis 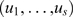 of an *s*-dimensional vector subspace 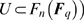 in general position with respect to the basis 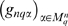. The next step is to calculate the variety
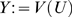
We assume 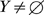 and order its elements arbitrarily to a tuple 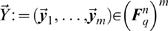, where 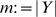. By (5) (see also Remark 23 in Subsection 4.3.2 of the Appendix S1) we know that
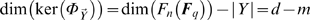



By the definitions we have in general
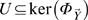
and therefore 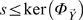, i.e. 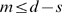. The ideal scenario would be the case 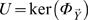, i.e. 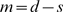. A less optimistic scenario is given when 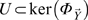. In such a situation, ideally we would wish for 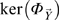 to be itself in general position with respect to the basis 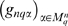. These issues raise the following question:

When does there exist a subspace 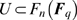 in general position with respect to the basis 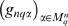 with 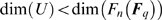 that in addition satisfies
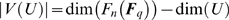
(8)


This is an interesting question that requires further research. It is related to whether the subspace *U* is an ideal of 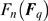 when 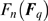 is seen as an algebra with the multiplication of polynomial functions as the multiplicative operation. In Section 2 of the Appendix S1 we provide examples in which two subspaces, both in general position, show a different behavior regarding the condition (8). We formalize this property:

**Definition 12** *For a given natural number* 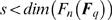, *let* 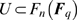 *be an s-dimensional subspace. U is said to satisfy the* codimension condition *if it holds*

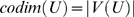

*where* 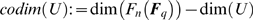.

A subspace 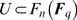 in general position with respect to the basis 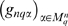 that satisfies the codimension condition allows for the construction of an optimal set for use with the LS-algorithm. The set 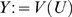 has namely the property 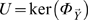, i.e. 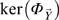 is in general position with respect to the basis 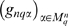. In other words, subspaces in general position that satisfy the codimension condition provide a fundamental component for a constructive method for generating optimal data sets. More generally we define:

**Definition 13** *A set* 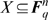 *such that* 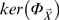 *is in general position with respect to the* 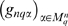 *is referred to as* optimal.

**Remark and Definition 14** *Additional study is required to prove whether optimal data sets exist in general. (See Section 2 of the* *Appendix S1* *for concrete examples.) However, if no optimal sets can be determined, it is still advantageous to work with a data set X that was obtained as* 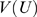 *using a subspace* 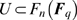 *in general position with respect to the basis* 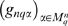. *In this case, at least* 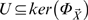 *still holds, and it might be that the dimensional difference between U and* 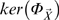 *is small. We call such data sets* pseudo-optimal.

### Probabilities of finding the original function as the orthogonal solution

In the previous subsection we were able to characterize optimal data sets based on a geometric property we called general position. The next step is to analyze the performance of the reverse engineering algorithms when such optimal data sets are used. In this subsection we specifically want to address the following two problems:

**Problem 15** *Let a function* 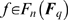 *and an optimal set* 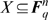 *of cardinality m be given. Furthermore let the values that f takes on every point in the set* 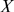 *be known. If the term order used by the LS-algorithm is chosen randomly, can the probability of successfully reconstructing f be calculated? If the linear order used by the term-order-free method is chosen randomly, can the probability of successfully reconstructing f be calculated?*

**Problem 16** *What is the asymptotic behavior of the probability for a growing number of variables n?*

The next theorem provides an answer to the second question stated in Problem 15 (regarding the term-order-free method):

**Theorem 17** *Let* 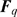 *be a finite field and* 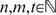 *natural numbers with* 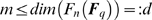 *and* 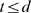. *Furthermore, let* 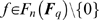 *be a nonzero polynomial function consisting of a linear combination of exactly t fundamental monomial functions. In addition, let* 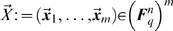 *be a tuple of m* ***different*** *n-tuples with entries in the field* 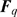 *such that* ***X is optimal*** *. Further, let* 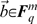 *be the vector defined by*

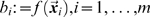


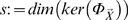
, 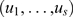 *a basis of* 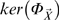 *and* 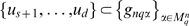 *an arbitrary subset containing* 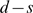 *elements. Then the probability P that the orthogonal solution* 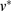 *of* 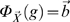 *with respect to the generalized inner product*

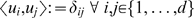

*fulfills* 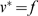 *is given by*

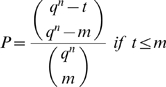
(9)
*and*

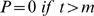



**Proof:** Due to the definition of general position, there are exactly
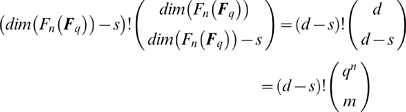
different ways to extend a basis 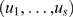 of *U* to a basis of 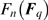 using 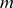 fundamental monomial functions. If 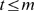, among such extensions, only
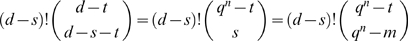
use the *t* fundamental monomial functions appearing in *f*. From this, (9) follows immediately. If, on the other hand, 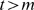, the number of fundamental monomial functions available to extend a basis 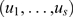 of *U* to a basis of 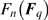 is too small.▪

**Remark 18** If the elements in the basis 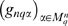 are ordered in a decreasing way according to a term order (the biggest element is at the left end, the smallest at the right end and position *y* means counting *y* elements from the right to the left) an analogous probability formula would be
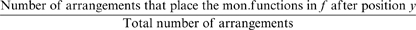
(10)where an arrangement is an order of the elements of 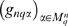 that obeys a term order. (Two different term orders could generate the same arrangement of the elements in the finite set 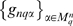) So, for instance, if *f* contains a term involving the monomial function 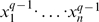, then the above probability (10) would be equal to zero, since every arrangement of the elements in 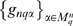 that obeys a term order would make this monomial function biggest. (It is inherent to term orders to make high degree monomial functions always biggest). In more general terms, it is difficult to make estimates about the numbers appearing in (10). How to calculate the above probability remains an open question.

**Remark 19** Since for relatively small *n* and *q* the number 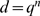 is already very large, it is obvious that one should calculate the asymptotic behavior of the probability formula (9) for 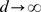. Indeed, we have with 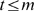


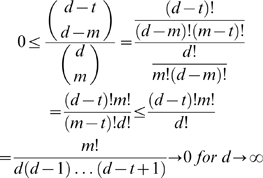



If we write the amount of data used in proportion to the size 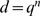 of the space 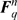 , and the number of terms displayed by *f* relative to the size 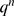 of the basis 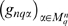, it becomes apparent how quickly the probability formula converges to *0* for 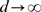. Accordingly, let 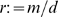 and 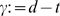. Then we would have
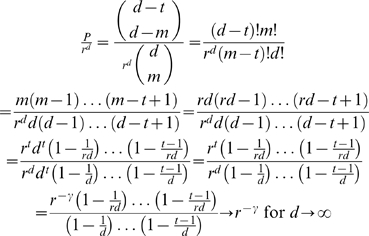



In particular, it holds
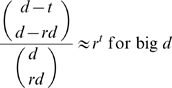
(11)This expression shows in a straightforward way how big the proportional amount of data should be in order to have an acceptable confidence in the result obtained. It also shows that for *t* close to *d*, the probability is very low and the reverse engineering not feasible. Usually no information about *t* is available, so it is advisable to work with the maximal *t*, namely 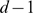 or with an average value for *t*.

For example, assume that in an experiment, *d* is sufficiently big and the average value for *t* is known and equal to 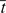. Furthermore, assume that one wants to reverse engineer a function 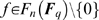 with a confidence 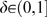 that the result is correct. The question is: How big should the cardinality *m* of an optimal data set *X* be (besides the necessary requirement 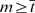)? According to (11), the requirement would be
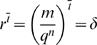
and therefore
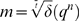



With elementary calculus it can be shown^13^ that if 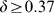 then
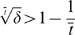
This lower bound for the proportion converges rapidly to *1* for increasing 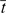. If 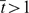, one can easily verify that
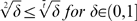



Thus, if the confidence 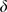 is to be greater or equal than *0.5*, then it holds
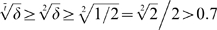



Consequently, if 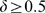 is required, already more than *70%* of the state space 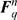 has to be sampled. Let us consider a relatively small biochemical network involving only *25* entities, where the concentrations of the entities can be meaningfully discretized to Boolean values *0* or *1*. In other words, *n = 25* and *q = 2*. The previous calculation tells us that more than 0.7 * 2^25^≈23.4 million experiments would be required.

**Example 20** *We provide a simple “academic” example, which, nevertheless, clearly presents the advantages of using optimal data sets and emphatically points out the issues related to the use of term orders. Assume n = 2 and q = 2. Thus, the space of functions we are dealing with is* 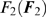. *The task is to reverse engineer the function*

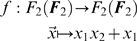

*Since the function f displays 2 terms, we need a data set containing at least 2 points in order to be able to completely reverse engineer f (see Theorem 6 and Remark 7). The next step is to try to find an optimal data set of cardinality at least 2. For this purpose, consider the basis* 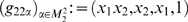 *of* 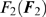 *and the one-dimensional vector subspace* 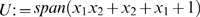. *The basis vector* 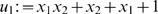 *has the coordinates* 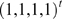 *with respect to the basis* 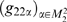. *Therefore, U is in general position with respect to* 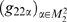 *(recall Definition 9). It is easy to verify*

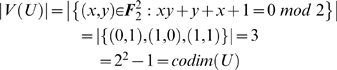



*As a consequence, the set* 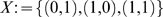 *constitutes an optimal data set (see Definitions 15 and 16) to reverse engineer any function* 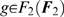 *displaying no more than 3 terms. According to (9), the probability of reconstructing f using the data set X and the term-order-free reverse engineering method with a randomly chosen linear order (for ordering the set* 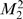 *) is*

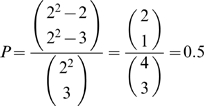

*However, if the data set X is used and only term orders are allowed (for ordering the set* 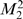 *), i.e. the LS-algorithm is used with X as input data, the probability of finding f would be equal to zero. This follows from the fact that for any term order, the term* 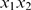 *is always the biggest. Note also that the term* 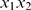 *does not vanish on X, in other words, that is not the reason why the LS-algorithm is unable to reverse engineer f. This is happening even though the data set X is relatively big, namely 75% of the entire state space* 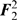. *A similar calculation (see Section 2 of the* *Appendix S1* *) shows that the reverse engineering of the function* 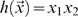 *would be successful with probability 0.75 using the term-order-free reverse engineering method fed with X, whereas the LS-algorithm (fed with X) could not find the correct function h*.

## Discussion

The results we have obtained in the previous section provide guidelines on how to design experiments to generate data to be used with the LS-algorithm for the purpose of reverse engineering a biochemical network.

The following are minimal requirements on a set 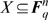, such that the LS-algorithm reverse engineers *f* based on the knowledge of the values that it takes on every point in the set *X*:

1. If the LS-algorithm is used to reverse engineer a nonzero function 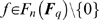, necessarily the data set *X* used must contain points were the function does not vanish. In other words, not all the interpolation conditions must be of the type 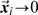 (Theorem 5).
2. If the LS-algorithm is used to reverse engineer a nonzero function 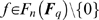 displaying *t* different terms, it requires **at least** *t* different data points to completely reverse engineer *f* (Remark 7).
3. If 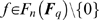 is a polynomial function containing all 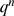 possible fundamental monomial functions, no proper subset 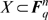 of 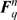 will allow the LS-algorithm to find *f* (Remark 7). 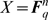 would do the job, however, as mentioned previously, experimental data are typically sparse.

Our results also make possible the identification of optimal sets 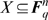 that make the LS-algorithm more likely to succeed in reverse engineering a function 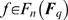 based only on the knowledge of the values that it takes on every point in the set *X*. Optimal data sets 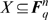 are characterized by the property that 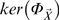 is in general position with respect to the basis 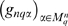 (see Definitions 16 and 12). Their advantage is given by the fact that they do not impose constraints on the set of candidate terms that can be used to construct a solution. Summarizing we can say:

1. Even though such sets can be constructed in particular examples (see Section 2 of the Appendix S1), further research is required to prove their existence in general terms.
2. If no optimal sets can be determined, it is still advantageous to work with pseudo-optimal data sets (see Remark and Definition 14).

Since the identified optimal data sets are sets 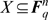 of discretized vectors, in a real application, the optimal data set *X* has to be transformed back (or “undiscretized”) to a corresponding set 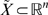 of real vectors. This transformation can be performed using an “inverse” function of the discretization mapping (1). This “inverse” function has to be defined by the user, given the fact that discretization mappings are highly non-injective^14^ and by definition map entire subsets 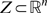 into a single value 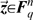. Once the set 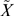 has been established, the experimental task is to measure how the system evolves from every state described by every single point in the set 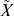, i.e. every point in 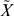 is used as initial conditions and the subsequent time evolution of the system is measured. This task is what we call the design of specific experiments. The criteria for this design are precisely the initial conditions to be used, which are provided by the set 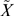, the “undiscretized” optimal data set 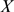.

Having characterized optimal data sets, the next step in our approach was to provide an exact formula for the probability that the LS-algorithm will find the correct model under the assumption that an optimal data set is used as input. As stated in Remark 18, we were not able to find such a formula for the LS-algorithm. The biggest difficulty we face is related to the use of term orders inherent to the LS-algorithm. We overcome this problem by considering a generalization of the LS-algorithm, the term-order-free reverse engineering method. This method not only allows for the calculation of the success probability but it also eliminates the issues and arbitrariness linked to the use of term orders (see Remark 18 and Example 20). In conclusion, our results on this issue are:

1. It is still an open problem how to derive a formula for the success probability of the LS-algorithm when optimal data sets are used as an input and the term order is chosen randomly. As stated in Remark 18, one of the main problems here is related to the use of term orders inherent to the LS-algorithm.
2. Let 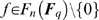 be a nonzero function consisting of the linear combination of exactly *t* fundamental monomial functions. If the linear order used by the term-order-free method is chosen randomly, the probability of successfully retrieving *f* using an optimal data set *X* of cardinality 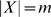 is given by (see Theorem 17)
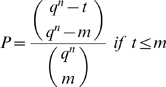
(12)and
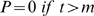

3. Let 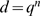 be the cardinality of the space 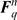. Furthermore, let *X* be an optimal data set with cardinality 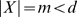 and 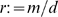 (note that 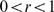). Then the asymptotic behavior of the probability formula (12) for 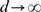 (i.e. for 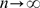) satisfies (see Remark 19)
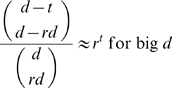

4. Let 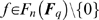 be as above. To reverse engineer *f* using the term-order-free method with a confidence 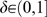, an optimal data set of cardinality 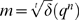 (provided 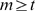) is required. Furthermore, for 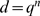 sufficiently big, it holds
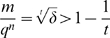
for the proportion of data points needed (see Remark 19).

As a consequence of the latter, we conclude that even if an optimal data set is used and the restrictions imposed by the use of term orders are overcome, the reverse engineering problem remains unfeasible, unless experimentally impracticable amounts of data are available.

At this point, it is pertinent to comment on one scenario identified in [11]. Specifically, in Conclusion 4(a), the author of [11] makes the assumption that the wiring diagram of each of the underlying functions is known, i.e. the variables that actually affect the function *f* are known. Under this assumption, let *k* be the number of variables affecting *f*. If one could perform specific experiments such that for all possible values that the *k* variables can take the response of the network is measured, the function *f* would be uniquely determined. In this situation, reverse engineering *f* would not imply making any choices among possible solutions. This raises the question of how many measurements are needed and how big this data set would be in proportion to the size 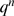 of the space 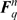 of all possible states the network can theoretically display. The number of measurements needed is 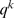 and therefore the proportion is equal to
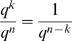
If *k* is small compared to *n* (which is generally assumed by the author of [11]), then the proportion would be conveniently small. In other words, in relative terms, it is worth performing the 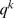 specific experiments. However, performing 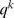 measurements might still be beyond experimental feasibility.

Reverse engineering within the modeling paradigm of time discrete finite dynamical systems requires the assumption that the state of the different entities modeled can be discretized in a meaningful way. Discretization is a challenging problem, which does not seem to have a universal solution. While discretization could help eliminate the noise in noisy data, it is by no means clear in general terms what should be considered noise and what a significant variation. Therefore, the threshold between noise and real variation has to be individually determined for every particular experimental setting.

Also the issue of choosing the number *q* of different discretized states represents a difficulty. As with any mathematical algorithmic method based on discretization, some type of convergence as the discretization gets finer and finer (i.e. the step size gets smaller) is highly desirable, in the sense that after a certain degree of resolution, the method is capable of catching essential properties which will not vary significantly if the resolution is further increased. We have partially explored the properties of the LS-algorithm in this regard. However, it would go beyond the scope of this paper to include our results here.

Since experimental measurements are discrete in time, a time discrete modeling approach seems natural. However, it is important to know the time scales of the different processes observed in order to use a frequency of measurement that will not miss important changes of the system. On the other hand, a measuring frequency that is too high could generate data that seem to report that the system observed has already reached a stable state.

Discretized data are also very rigid in the sense that it is not easy to establish what the neighborhood of a point could be. It would be namely interesting to study, how small perturbations in the discrete input data are propagated in the LS-algorithm and how the output model responds to those perturbations. To do this mathematically, one would need to introduce a topological structure in the state space 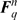 as well as in the function space 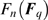. Since the LS-algorithm is based on exact interpolation, we expect the effects of perturbation in the data set to be ill-conditioned. However, we are not able to express this sensitivity to perturbations in the input data in a systematic way. The main reason for this is that our explorations of this issue have not yielded any helpful way to define a topological structure that would capture a meaningful notion of neighborhood. This failure seems to be closely related to the discrete character of these spaces.

In this sense, a state continuous modeling paradigm seems to be significantly more tractable from a topological point of view.

Recent developments in applied commutative algebra and computational algebraic geometry have proposed the use of generalized normal forms [23] and normal forms with respect to border bases [24], [25]. These developments generalize Gröbner bases approaches by dropping the requirement for term orders. In the light of these developments, the question arises as to what extent the LS-algorithm could advantageously be adapted to the use of these more general types of normal forms. The feasibility of such an adaption as well as its computational aspects remain to be investigated.

### Endnotes

^1^ A finite field is a finite nonempty set endowed with the algebraic structure of a field, i.e. operations of addition and multiplication of pairs of elements are defined and follow precise rules. The simplest finite field is the Boolean field 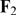 which only contains the elements 0 and 1.

^2^ The upper bound *q* for the degree results from the algebraic fact that 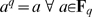.

^3^ A linear operator 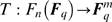 is a function that preserves the vector space structure, i.e. linear combinations are mapped into linear combinations. Surjective means that for every 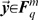 there is a 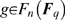 such that 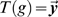. A surjective linear operator is called epimorphism.

^4^ For a given set 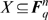 we construct the tuple 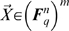 by ordering the elements of 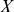 according to a fixed arbitrary order.

^5^ 
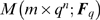
 is the ring of 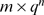 matrices with entries in 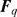.

^6^ The kernel 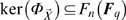 of 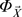 is the subspace of 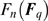 containing all the functions *g* with the property 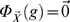.

^7^ A bilinear form is a mapping that takes two vectors and maps them into the underlying field in a way such that the mapping is linear in each of its arguments. Such a bilinear form is called symmetric if interchanging the arguments does not alter the value of the mapping.

^8^ 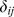 is the Kronecker Delta, equal to one for equal indices and otherwise equal to zero.

^9^ As an example, this is how the standard inner product in the real vector space 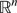 is defined, where the basis vectors are the canonical unit vectors 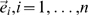.

^10^ A (strict) partial order < on a set S is a nonreflexive, antisymmetric and transitive binary relation.

^11^ The vector 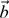 is obtained from the measurements or simulations.

^12^ If 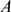 is a set, 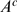 denotes its complement.

^13^ This follows from the fact 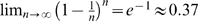.

^14^ A function 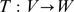 from set *V* to set *W* is called injective if 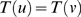 implies 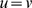.

## Supporting Information

Appendix S1 Examples and technical proofs.
(0.21 MB PDF)
