# Supplementary material for: Reverse Engineering Time Discrete Finite Dynamical Systems: A Feasible Undertaking?
Source: PLoS One. 2009 Mar 19;4(3):e4939. doi: 10.1371/journal.pone.0004939 (PMC2654658; doi:10.1371/journal.pone.0004939)
Supplement: Appendix S1 — Examples and technical proofs. (0.21 MB PDF) [file pone.0004939.s001.pdf]

Appendix

1 The algorithm in action: A simple example

Example 1 *Let $n = 3$, $q = 3$ and consider the $d := 3^3 = 27$-dimensional vector space $F_3(\mathbf{F}_3)$. The task is to reverse engineer the function $f \in F_3(\mathbf{F}_3)$ defined as*

$$\begin{aligned} f &: \mathbf{F}_3^3 \to \mathbf{F}_3 \\ \vec{x} &\mapsto x_1^2x_2^2x_3^2 + 2x_1^2 + 2x_2 + x_3 + 2 \end{aligned}$$

based on the knowledge of the values it takes on every of the nine points (i.e. $m = 9$) in the set

$$X := \{(0,0,1),(0,1,0),(0,1,2),(1,0,2),(1,1,0),(1,1,2),(2,1,1),(2,2,1),(2,2,2)\}$$

which are $\vec{b} := (0,1,0,0,0,0,2,1,2)$. As shown in the Methods Section, the term-order-free reverse engineering method fed with a term order (and with X) generates the same solution that the LS-algorithm would calculate using the same term order and X as its input. If the term-order-free reverse engineering method is fed with a more general linear order, it generates a solution that the LS-algorithm might not be able to produce (cf. Example 23 in the Results Section). To illustrate how the term-order-free reverse engineering method works in both cases, we consider two order relations: On the one hand, the lexicographic order $<_{lex}$with $x_1 > x_2 > x_3$ and, on the other hand, a fixed but arbitrary linear order $<_{lin}$. In other words, we need to order the basis $(g_{33\alpha})_{\alpha \in M_3^3}$ of the space $F_3(\mathbf{F}_3)$ according to both order relations. This yields

$$\begin{aligned} (g_{33\alpha})_{\alpha \in M_3^3(<_{lex})} = \; & (x_1^2x_2^2x_3^2, x_1^2x_2^2x_3, x_1^2x_2^2, x_1^2x_2x_3^2, x_1^2x_2x_3, x_1^2x_2, x_1^2x_3^2, x_1^2x_3, x_1^2, x_1x_2^2x_3^2, x_1x_2^2x_3, \\ & x_1x_2^2, x_1x_2x_3^2, x_1x_2x_3, x_1x_2, x_1x_3^2, x_1x_3, x_1, x_2^2x_3^2, x_2^2x_3, x_2^2, x_2x_3^2, x_2x_3, x_2, x_3^2, x_3, 1) \end{aligned}$$

and (for instance)

$$\begin{aligned} (g_{33\alpha})_{\alpha \in M_3^3(<_{lin})} = \; & (1, x_2^2x_3, x_1^2x_2x_3^2, x_1^2x_3, x_2^2x_3^2, x_2, x_1x_2x_3^2, x_2x_3, x_1x_3, x_3^2, x_1^2x_2, x_2x_3^2, x_1^2x_2^2x_3, x_1x_3^2, \\ & x_1x_2^2x_3^2, x_1, x_1x_2^2, x_1^2x_2^2, x_3, x_2^2, x_1x_2, x_1x_2^2x_3, x_1x_2x_3, x_1^2, x_1^2x_2x_3, x_1^2x_3^2, x_1^2x_2^2x_3^2) \end{aligned}$$

Consequently, the matrix $A(<_{lex}) := (\Phi_{\vec{X}}(g_{33\alpha}))_{\alpha \in M_3^3(<_{lex})} \in M(9 \times 3^3; \mathbf{F}_3)$ representing the evaluation epimorphism with respect to the basis $(g_{33\alpha})_{\alpha \in M_3^3(<_{lex})}$ is given by

$$A(<_{lex}) = \begin{pmatrix} 0,0,0,0,0,0,0,0,0,0,0,0,0,0,0,0,0,0,0,0,0,0,0,0,1,1,1 \\ 0,0,0,0,0,0,0,0,0,0,0,0,0,0,0,0,0,0,0,0,1,0,0,1,0,0,1 \\ 0,0,0,0,0,0,0,0,0,0,0,0,0,0,0,0,0,0,1,2,1,1,2,1,1,2,1 \\ 0,0,0,0,0,0,1,2,1,0,0,0,0,0,0,1,2,1,0,0,0,0,0,0,1,2,1 \\ 0,0,1,0,0,1,0,0,1,0,0,1,0,0,1,0,0,1,0,0,1,0,0,1,0,0,1 \\ 1,2,1,1,2,1,1,2,1,1,2,1,1,2,1,1,2,1,1,2,1,1,2,1,1,2,1 \\ 1,1,1,1,1,1,1,1,1,2,2,2,2,2,2,2,2,2,1,1,1,1,1,1,1,1,1 \\ 1,1,1,2,2,2,1,1,1,2,2,2,1,1,1,2,2,2,1,1,1,2,2,2,1,1,1 \\ 1,2,1,2,1,2,1,2,1,2,1,2,1,2,1,2,1,2,1,2,1,2,1,2,1,2,1 \end{pmatrix}$$

and with respect to the basis $(g_{33\alpha})_{\alpha \in M_3^3(<_{lin})}$ by

$$A(<_{lin}) = \begin{pmatrix} 1,0,0,0,0,0,0,0,0,1,0,0,0,0,0,0,0,0,1,0,0,0,0,0,0,0,0 \\ 1,0,0,0,0,1,0,0,0,0,0,0,0,0,0,0,0,0,1,0,0,0,0,0,0,0,0 \\ 1,2,0,0,1,1,0,2,0,1,0,1,0,0,0,0,0,0,2,1,0,0,0,0,0,0,0 \\ 1,0,0,2,0,0,0,0,2,1,0,0,0,1,0,1,0,0,2,0,0,0,0,1,0,1,0 \\ 1,0,0,0,0,1,0,0,0,0,1,0,0,0,0,1,1,1,0,1,1,0,0,1,0,0,0 \\ 1,2,1,2,1,1,1,2,2,1,1,1,2,1,1,1,1,1,2,1,1,2,2,1,2,1,1 \\ 1,1,1,1,1,1,2,1,2,1,1,1,1,2,2,2,2,1,1,1,2,2,2,1,1,1,1 \\ 1,1,2,1,1,2,1,2,2,1,2,2,1,2,2,2,2,1,1,1,1,2,1,1,2,1,1 \\ 1,2,2,2,1,2,1,1,1,1,2,2,2,2,2,2,2,1,2,1,1,1,2,1,1,1,1 \end{pmatrix}$$

It can be easily verified that the rank of the above matrices is equal to 9*. Thus* $s := \dim(Ker(\Phi_{\vec{X}})) = 27 - 9 = 18$*. The next step in our method is to calculate a basis of* $Ker(\Phi_{\vec{X}})$*. Coordinate vectors* $\vec{y}_1, ..., \vec{y}_{18}$ *(with respect to the basis* $(g_{33\alpha})_{\alpha \in M_3^3(\cdot)}$*) of a basis are obtained through Gaussian elimination on the matrix A. If we write each coordinate vector* $\vec{y}_i$ *as a row in a matrix we obtain*

$$B(<_{lex}) = \begin{pmatrix}
0,0,0,0,0,0,0,0,0,0,0,0,0,0,0,0,0,0,1,0,2,0,0,0,2,0,1 \\
0,2,0,0,0,0,2,0,0,0,0,0,0,0,0,0,0,0,2,0,0,0,0,0,2,1,0 \\
0,2,0,1,2,0,2,0,0,0,0,0,0,0,0,1,0,0,0,0,0,0,0,0,0,0,0 \\
0,2,2,1,2,0,0,0,0,0,0,1,0,0,0,0,0,0,0,0,0,0,0,0,0,0,0 \\
1,0,0,2,0,0,0,0,0,0,0,0,0,0,0,0,0,0,0,0,2,0,0,1,0,0,0 \\
1,0,0,2,0,0,0,0,0,0,0,0,0,0,0,0,0,0,2,0,0,1,0,0,0,0,0 \\
1,0,2,0,0,0,2,0,1,0,0,0,0,0,0,0,0,0,0,0,0,0,0,0,0,0,0 \\
1,0,2,2,0,1,0,0,0,0,0,0,0,0,0,0,0,0,0,0,0,0,0,0,0,0,0 \\
1,2,0,2,1,0,1,0,0,0,0,0,0,0,0,0,1,0,0,0,0,0,0,0,0,0,0 \\
1,2,0,2,2,0,0,0,0,0,0,0,1,0,0,0,0,0,0,0,0,0,0,0,0,0,0 \\
1,2,2,1,2,0,2,0,0,0,0,0,0,0,0,0,0,1,0,0,0,0,0,0,0,0,0 \\
2,0,0,0,2,0,0,0,0,0,0,0,0,0,0,0,0,0,1,0,0,0,1,0,0,0,0 \\
2,1,0,2,2,0,0,0,0,0,0,0,0,1,0,0,0,0,0,0,0,0,0,0,0,0,0 \\
2,2,0,0,0,0,0,0,0,0,0,0,0,0,0,0,0,0,1,1,0,0,0,0,0,0,0 \\
2,2,0,0,0,0,1,1,0,0,0,0,0,0,0,0,0,0,0,0,0,0,0,0,0,0,0 \\
2,2,0,1,2,0,0,0,0,1,0,0,0,0,0,0,0,0,0,0,0,0,0,0,0,0,0 \\
2,2,0,2,1,0,0,0,0,0,1,0,0,0,0,0,0,0,0,0,0,0,0,0,0,0,0 \\
2,2,2,2,2,0,0,0,0,0,0,0,0,0,1,0,0,0,0,0,0,0,0,0,0,0,0
\end{pmatrix}$$

and

$$B(<_{lin}) = \begin{pmatrix}
0,0,1,0,0,0,2,0,0,0,2,0,0,0,0,0,0,0,0,0,1,0,0,0,0,0,0 \\
0,0,2,1,0,0,2,0,2,0,0,0,1,0,0,0,0,0,0,0,0,0,0,0,0,0,0 \\
0,0,2,2,0,0,2,0,1,0,0,0,0,0,0,0,0,0,0,0,1,0,0,0,0,0,0 \\
0,1,0,0,1,0,0,0,1,0,0,0,0,1,0,0,0,0,0,0,0,0,0,0,0,0,0 \\
0,1,1,0,1,0,0,0,1,0,2,0,0,0,0,1,0,0,0,0,0,0,0,0,0,0,0 \\
0,1,1,0,2,0,2,1,0,0,0,0,0,0,0,0,0,0,0,0,0,0,0,0,0,0,0 \\
0,1,1,1,0,0,2,0,2,0,0,1,0,0,0,0,0,0,0,0,0,0,0,0,0,0,0 \\
0,1,1,1,1,0,1,0,2,0,0,0,0,0,1,0,0,0,0,0,0,0,0,0,0,0,0 \\
0,1,1,2,1,0,0,0,1,0,0,0,0,0,0,0,0,0,0,0,0,1,0,0,0,0,0 \\
0,1,2,1,1,0,1,0,2,0,2,0,0,0,0,0,1,0,0,0,0,0,0,0,0,0,0 \\
0,2,0,1,2,0,0,0,0,0,0,0,0,0,0,0,0,0,0,0,0,0,0,0,0,1,0 \\
0,2,0,1,2,0,1,0,2,0,0,0,0,0,0,0,0,0,0,0,0,0,0,0,1,0,0 \\
0,2,1,1,2,0,0,0,0,0,2,0,0,0,0,0,0,0,0,0,0,0,0,1,0,0,0 \\
0,2,1,2,2,0,1,0,1,0,0,0,0,0,0,0,0,0,0,0,0,0,0,0,0,0,1 \\
0,2,2,2,2,0,1,0,1,0,2,0,0,0,0,0,0,1,0,0,0,0,0,0,0,0,0 \\
0,2,2,2,2,2,1,0,1,0,0,0,0,0,0,0,0,0,0,1,0,0,0,0,0,0,0 \\
2,0,0,0,1,1,1,0,1,0,0,0,0,0,0,0,0,0,1,0,0,0,0,0,0,0,0 \\
2,1,1,1,0,1,2,0,2,1,0,0,0,0,0,0,0,0,0,0,0,0,0,0,0,0,0
\end{pmatrix}$$

To extend this basis we apply the standard orthonormalization procedure, which begins by performing Gaussian elimination on the matrices above. We get

$$\widetilde{B}(<_{lex}) = \begin{pmatrix}
1,0,0,2,0,0,0,0,0,0,0,0,0,0,0,0,0,0,0,0,2,0,0,1,0,0,0 \\
0,1,0,0,0,0,1,0,0,0,0,0,0,0,0,0,0,0,1,0,0,0,0,0,1,2,0 \\
0,0,1,2,1,0,2,0,0,0,0,2,0,0,0,0,0,0,2,0,0,0,0,0,2,1,0 \\
0,0,0,1,2,0,0,0,0,0,0,0,0,0,0,1,0,0,1,0,0,0,0,0,1,2,0 \\
0,0,0,0,1,0,1,0,1,0,0,2,0,0,0,0,0,0,2,0,1,0,0,2,2,1,0 \\
0,0,0,0,0,1,2,0,0,0,0,2,0,0,0,1,0,0,0,0,1,0,0,2,0,0,0 \\
0,0,0,0,0,0,1,0,2,0,0,1,0,0,0,0,1,0,2,0,0,0,0,0,2,1,0 \\
0,0,0,0,0,0,0,1,1,0,0,2,0,0,0,1,0,0,1,0,0,0,0,0,1,2,0 \\
0,0,0,0,0,0,0,0,1,0,0,2,0,0,0,1,1,0,2,0,1,0,1,2,1,2,0 \\
0,0,0,0,0,0,0,0,0,1,0,0,0,0,0,0,1,0,2,0,0,0,0,0,2,1,0 \\
0,0,0,0,0,0,0,0,0,0,1,0,0,0,0,2,1,0,1,0,0,0,0,0,1,2,0 \\
0,0,0,0,0,0,0,0,0,0,0,1,0,0,0,1,1,2,2,0,0,0,0,0,2,1,0 \\
0,0,0,0,0,0,0,0,0,0,0,0,1,0,0,0,1,0,2,0,2,0,0,1,2,1,0 \\
0,0,0,0,0,0,0,0,0,0,0,0,0,1,0,0,2,0,2,0,0,0,1,0,1,2,0 \\
0,0,0,0,0,0,0,0,0,0,0,0,0,0,1,1,1,2,2,0,2,0,0,1,2,1,0 \\
0,0,0,0,0,0,0,0,0,0,0,0,0,0,0,1,1,0,1,1,0,0,0,0,0,0,0 \\
0,0,0,0,0,0,0,0,0,0,0,0,0,0,0,0,0,0,1,0,2,0,0,0,2,0,1 \\
0,0,0,0,0,0,0,0,0,0,0,0,0,0,0,0,0,0,0,0,0,1,0,2,2,0,1
\end{pmatrix}$$

and

$$\widetilde{B}(<_{lin}) = \begin{pmatrix}
1,0,0,0,2,2,2,0,2,0,0,0,0,0,0,0,0,0,2,0,0,0,0,0,0,0,0 \\
0,1,0,0,1,0,0,0,1,0,0,0,0,1,0,0,0,0,0,0,0,0,0,0,0,0,0 \\
0,0,1,1,0,0,1,0,2,0,0,0,0,0,0,0,0,0,0,0,2,0,0,0,0,0,0 \\
0,0,0,1,0,0,0,0,2,0,0,0,2,0,0,0,0,0,0,0,1,0,0,0,0,0,0 \\
0,0,0,0,1,0,1,1,2,0,0,0,2,2,0,0,0,0,0,0,2,0,0,0,0,0,0 \\
0,0,0,0,0,1,1,0,2,0,0,0,0,2,0,0,0,0,0,2,0,1,0,0,0,0,0 \\
0,0,0,0,0,0,1,2,2,0,0,2,1,2,0,0,0,0,0,0,0,0,0,0,0,0,0 \\
0,0,0,0,0,0,0,1,2,0,0,1,1,2,0,0,0,0,0,0,0,0,2,0,0,0,0 \\
0,0,0,0,0,0,0,0,1,0,0,0,0,1,2,0,0,0,0,0,0,2,0,0,0,0,0 \\
0,0,0,0,0,0,0,0,0,1,0,1,1,1,2,0,0,0,2,0,0,1,1,0,0,0,0 \\
0,0,0,0,0,0,0,0,0,0,1,0,1,1,0,0,0,0,0,0,0,2,1,2,0,0,0 \\
0,0,0,0,0,0,0,0,0,0,0,0,1,0,0,0,0,0,0,0,0,1,2,0,2,0,0 \\
0,0,0,0,0,0,0,0,0,0,0,0,0,1,1,0,0,0,0,0,0,0,2,0,2,2,0 \\
0,0,0,0,0,0,0,0,0,0,0,0,0,0,1,0,0,0,0,0,0,0,1,0,1,0,1 \\
0,0,0,0,0,0,0,0,0,0,0,0,0,0,0,1,0,0,0,0,0,0,1,2,1,0,2 \\
0,0,0,0,0,0,0,0,0,0,0,0,0,0,0,0,1,0,0,0,0,0,1,2,1,1,1 \\
0,0,0,0,0,0,0,0,0,0,0,0,0,0,0,0,0,1,0,0,0,0,0,2,0,1,2 \\
0,0,0,0,0,0,0,0,0,0,0,0,0,0,0,0,0,0,0,0,1,2,0,2,1,1,2
\end{pmatrix}$$

The standard orthonormalization procedure extends the basis based on the columns within which the Gaussian elimination could not find a pivot element. For instace, in the case of $\widetilde{B}(<_{lin})$*, the colums* $12, 19, 20, 22, 23, ..., 27$ *lack a pivot element. Therefore, the standard orthonormalization procedure extends the coordinate vectors* $\vec{y}_1, ..., \vec{y}_{18}$ *of the basis by including the canonical unit vectors* $\vec{e}_{12}, \vec{e}_{19}, \vec{e}_{20}, \vec{e}_{22}, \vec{e}_{23}, ..., \vec{e}_{27}$*. If we write the*

coordinate vectors of the new basis as the columns of a matrix we get

$$Y(<_{lex}) = \begin{pmatrix}
2,1,2,1,0,1,2,2,2,2,1,0,1,0,1,2,0,0,1,0,0,0,0,0,0,0,0 \\
2,2,2,2,2,2,1,2,0,2,0,2,0,2,0,2,0,0,0,0,0,0,0,0,0,0,0 \\
0,0,0,0,2,2,0,2,0,0,2,0,0,0,2,0,0,0,0,0,0,0,0,0,0,0,0 \\
0,2,0,2,1,1,2,2,0,1,0,1,2,0,2,2,0,0,2,0,0,0,0,0,0,0,0 \\
0,2,0,1,2,2,2,2,2,2,0,2,0,0,0,1,0,0,0,0,0,0,0,0,0,0,0 \\
0,0,0,0,0,0,0,0,0,0,0,0,0,0,1,0,0,0,0,0,0,0,0,0,0,0,0 \\
1,0,0,1,0,2,0,0,0,0,2,2,0,2,0,0,0,0,0,0,0,0,0,0,0,0,0 \\
1,0,0,0,0,0,0,0,0,0,0,0,0,0,0,0,0,0,0,0,0,0,0,0,0,0,0 \\
0,0,0,0,0,0,0,0,0,0,1,0,0,0,0,0,0,0,0,0,0,0,0,0,0,0,0 \\
0,0,0,0,0,0,0,0,0,1,0,0,0,0,0,0,0,0,0,0,0,0,0,0,0,0,0 \\
0,0,0,0,0,0,0,0,0,0,0,0,0,0,0,1,0,0,0,0,0,0,0,0,0,0,0 \\
0,0,0,0,1,0,0,0,0,0,0,0,0,0,0,0,0,0,0,0,0,0,0,0,0,0,0 \\
0,1,0,0,0,0,0,0,0,0,0,0,0,0,0,0,0,0,0,0,0,0,0,0,0,0,0 \\
0,0,0,0,0,0,1,0,0,0,0,0,0,0,0,0,0,0,0,0,0,0,0,0,0,0,0 \\
0,0,0,0,0,0,0,1,0,0,0,0,0,0,0,0,0,0,0,0,0,0,0,0,0,0,0 \\
0,0,0,0,0,0,0,0,0,0,0,1,0,0,0,0,0,0,0,0,0,0,0,0,0,0,0 \\
0,0,0,1,0,0,0,0,0,0,0,0,0,0,0,0,1,0,0,0,0,0,0,0,0,0,0 \\
0,0,0,0,0,1,0,0,0,0,0,0,0,0,0,0,0,1,0,0,0,0,0,0,0,0,0 \\
0,0,1,0,0,0,0,0,1,0,0,0,0,2,0,0,0,0,2,0,0,1,0,0,0,0,0 \\
0,0,1,0,0,0,0,0,0,0,0,0,0,0,0,0,0,0,0,1,0,0,0,0,0,0,0 \\
0,0,0,0,0,0,0,0,0,0,0,0,2,0,0,0,0,0,0,0,1,2,0,0,0,0,0 \\
0,0,0,0,0,0,0,0,0,0,0,0,0,0,0,0,0,0,1,0,0,0,0,0,0,0,0 \\
0,0,0,0,0,0,0,0,1,0,0,0,0,0,0,0,0,0,0,0,0,0,1,0,0,0,0 \\
0,0,0,0,0,0,0,0,0,0,0,0,1,0,0,0,0,0,0,0,0,0,0,1,0,0,0 \\
0,0,0,0,0,0,0,0,0,0,0,0,0,2,0,0,0,0,0,0,0,2,0,0,1,0,0 \\
0,0,0,0,0,0,0,0,0,0,0,0,0,1,0,0,0,0,0,0,0,0,0,0,0,1,0 \\
0,0,0,0,0,0,0,0,0,0,0,0,0,0,0,0,0,0,0,0,0,1,0,0,0,0,1
\end{pmatrix}$$

and

$$
Y(<_{lin}) = \begin{pmatrix}
0, 2, 0, 0, 0, 0, 0, 0, 0, 2, 0, 0, 0, 0, 0, 0, 0, 0, 0, 0, 0, 0, 0, 0, 0, 0, 0 \\
1, 1, 1, 0, 1, 1, 1, 1, 2, 0, 2, 0, 0, 0, 1, 2, 2, 2, 0, 0, 2, 0, 0, 0, 0, 0, 0 \\
1, 1, 1, 2, 0, 1, 1, 2, 2, 0, 2, 0, 1, 2, 1, 1, 0, 0, 0, 0, 1, 0, 0, 0, 0, 0, 0 \\
0, 1, 1, 1, 0, 1, 0, 1, 2, 0, 2, 0, 0, 2, 2, 1, 1, 1, 0, 0, 2, 0, 0, 0, 0, 0, 0 \\
2, 0, 0, 0, 1, 1, 1, 1, 2, 1, 2, 0, 0, 0, 1, 2, 2, 2, 0, 0, 2, 0, 0, 0, 0, 0, 0 \\
0, 1, 0, 0, 0, 0, 0, 0, 0, 1, 2, 0, 0, 0, 0, 0, 0, 0, 0, 0, 0, 0, 0, 0, 0, 0, 0 \\
2, 2, 2, 2, 0, 1, 0, 1, 1, 1, 1, 0, 2, 2, 0, 0, 1, 0, 0, 0, 1, 0, 0, 0, 0, 0, 0 \\
1, 0, 0, 0, 0, 0, 0, 0, 0, 0, 0, 0, 0, 0, 0, 0, 0, 0, 0, 0, 0, 0, 0, 0, 0, 0, 0 \\
0, 2, 2, 2, 1, 2, 1, 2, 1, 1, 1, 0, 0, 1, 1, 0, 2, 0, 0, 0, 1, 0, 0, 0, 0, 0, 0 \\
0, 1, 0, 0, 0, 0, 0, 0, 0, 0, 0, 0, 0, 0, 0, 0, 0, 0, 0, 0, 0, 0, 0, 0, 0, 0, 0 \\
0, 0, 0, 0, 0, 0, 2, 2, 2, 0, 0, 0, 2, 0, 0, 2, 0, 0, 0, 0, 0, 0, 0, 0, 0, 0, 0 \\
0, 0, 1, 0, 0, 0, 0, 0, 0, 0, 0, 1, 0, 0, 0, 0, 0, 0, 0, 0, 0, 0, 0, 0, 0, 0, 0 \\
0, 0, 0, 1, 0, 0, 0, 0, 0, 0, 0, 0, 0, 0, 0, 0, 0, 0, 0, 0, 0, 0, 0, 0, 0, 0, 0 \\
0, 0, 0, 0, 1, 0, 0, 0, 0, 0, 0, 0, 0, 0, 0, 0, 0, 0, 0, 0, 0, 0, 0, 0, 0, 0, 0 \\
0, 0, 0, 0, 0, 1, 0, 0, 0, 0, 0, 0, 0, 0, 0, 0, 0, 0, 0, 0, 0, 0, 0, 0, 0, 0, 0 \\
0, 0, 0, 0, 0, 0, 1, 0, 0, 0, 0, 0, 0, 0, 0, 0, 0, 0, 0, 0, 0, 0, 0, 0, 0, 0, 0 \\
0, 0, 0, 0, 0, 0, 0, 1, 0, 0, 0, 0, 0, 0, 0, 0, 0, 0, 0, 0, 0, 0, 0, 0, 0, 0, 0 \\
0, 0, 0, 0, 0, 0, 0, 0, 1, 0, 0, 0, 0, 0, 0, 0, 0, 0, 0, 0, 0, 0, 0, 0, 0, 0, 0 \\
0, 0, 0, 0, 0, 0, 0, 0, 0, 1, 0, 0, 0, 0, 0, 0, 0, 0, 1, 0, 0, 0, 0, 0, 0, 0, 0 \\
0, 0, 0, 0, 0, 0, 0, 0, 0, 0, 1, 0, 0, 0, 0, 0, 0, 0, 0, 1, 0, 0, 0, 0, 0, 0, 0 \\
0, 0, 0, 0, 0, 0, 0, 0, 0, 0, 0, 0, 1, 0, 0, 0, 0, 0, 0, 0, 0, 0, 0, 0, 0, 0, 0 \\
0, 0, 0, 0, 0, 0, 0, 0, 0, 0, 0, 0, 0, 1, 0, 0, 0, 0, 0, 0, 0, 1, 0, 0, 0, 0, 0 \\
0, 0, 0, 0, 0, 0, 0, 0, 0, 0, 0, 0, 0, 0, 1, 0, 0, 0, 0, 0, 0, 0, 1, 0, 0, 0, 0 \\
0, 0, 0, 0, 0, 0, 0, 0, 0, 0, 0, 0, 0, 0, 0, 1, 0, 0, 0, 0, 0, 0, 0, 1, 0, 0, 0 \\
0, 0, 0, 0, 0, 0, 0, 0, 0, 0, 0, 0, 0, 0, 0, 0, 1, 0, 0, 0, 0, 0, 0, 0, 1, 0, 0 \\
0, 0, 0, 0, 0, 0, 0, 0, 0, 0, 0, 0, 0, 0, 0, 0, 0, 1, 0, 0, 0, 0, 0, 0, 0, 1, 0 \\
0, 0, 0, 0, 0, 0, 0, 0, 0, 0, 0, 0, 0, 0, 0, 0, 0, 0, 0, 0, 1, 0, 0, 0, 0, 0, 1
\end{pmatrix}
$$

Since the definition of the generalized inner product (as defined in Step 4 of the term-order-free reverse engineering method) holds for all $i, j \in \{1, ..., n\}$*, it follows for the matrix* S *(see Step 4 on page 12 of the main article)* $S = (Y^{-1})^t Y^{-1}$*, where* Y *is the matrix whose columns correspond to the coordinate vectors of*

the basis obtained from the standard orthonormalization procedure. Thus, we have

$$S(<_{lex}) = \begin{pmatrix}
1,2,2,2,2,0,0,0,1,0,0,1,2,0,0,2,2,0,2,2,1,0,2,1,0,2,1 \\
2,1,1,0,2,2,1,2,0,2,1,1,1,0,0,1,1,0,2,2,0,0,1,2,1,1,1 \\
2,1,1,2,2,1,2,1,1,0,0,2,0,2,2,0,0,1,0,0,2,0,2,1,1,2,0 \\
2,0,2,0,0,0,0,2,0,2,2,2,1,2,1,0,2,0,1,2,0,2,2,2,0,2,1 \\
2,2,2,0,1,0,0,1,0,2,0,2,1,1,1,0,1,0,2,1,0,0,2,2,1,1,1 \\
0,2,1,0,0,2,2,0,0,2,2,0,2,1,0,1,0,1,2,0,1,2,2,2,1,2,0 \\
0,1,2,0,0,2,0,1,2,1,1,0,1,2,0,1,2,0,2,1,2,2,2,1,0,0,0 \\
0,2,1,2,1,0,1,2,2,1,0,2,2,1,0,2,1,0,2,0,2,1,1,2,1,0,2 \\
1,0,1,0,0,0,2,2,0,1,1,1,2,1,2,2,0,1,0,2,0,2,2,1,1,0,2 \\
0,2,0,2,2,2,1,1,1,0,2,2,0,2,0,0,2,0,2,0,1,1,0,0,2,2,2 \\
0,1,0,2,0,2,1,0,1,2,1,2,0,1,0,0,1,0,0,2,1,2,0,0,0,1,2 \\
1,1,2,2,2,0,0,2,1,2,2,1,1,1,2,1,0,1,0,1,2,1,0,0,0,2,1 \\
2,1,0,1,1,2,1,2,2,0,0,1,2,0,2,2,2,0,0,0,2,2,0,2,2,2,2 \\
0,0,2,2,1,1,2,1,1,2,1,1,0,1,2,1,1,0,0,0,1,2,2,0,0,1,2 \\
0,0,2,1,1,0,0,0,2,0,0,2,2,2,2,0,0,1,1,1,0,2,0,2,0,2,1 \\
2,1,0,0,0,1,1,2,2,0,0,1,2,1,0,0,2,0,2,2,2,0,0,0,2,0,1 \\
2,1,0,2,1,0,2,1,0,2,1,0,2,1,0,2,1,0,0,0,0,0,0,0,0,0,0 \\
0,0,1,0,0,1,0,0,1,0,0,1,0,0,1,0,0,1,0,0,0,0,0,0,0,0,0 \\
2,2,0,1,2,2,2,2,0,2,0,0,0,0,1,2,0,0,1,0,1,0,0,0,1,0,2 \\
2,2,0,2,1,0,1,0,2,0,2,1,0,0,1,2,0,0,0,1,0,0,0,0,0,0,0 \\
1,0,2,0,0,1,2,2,0,1,1,2,2,1,0,2,0,0,1,0,1,0,0,0,0,0,0 \\
0,0,0,2,0,2,2,1,2,1,2,1,2,2,2,0,0,0,0,0,0,0,0,1,1,0,2 \\
2,1,2,2,2,2,2,1,2,0,0,0,0,2,0,0,0,0,0,0,0,0,1,0,0,0,0 \\
1,2,1,2,2,2,1,2,1,0,0,0,2,0,2,0,0,0,0,0,0,1,0,1,0,0,0 \\
0,1,1,0,1,1,0,1,1,2,0,0,2,0,0,2,0,0,1,0,0,1,0,0,1,0,0 \\
2,1,2,2,1,2,0,0,0,2,1,2,2,1,2,0,0,0,0,0,0,0,0,0,0,1,0 \\
1,1,0,1,1,0,0,2,2,2,2,1,2,2,1,1,0,0,2,0,0,2,0,0,0,0,1
\end{pmatrix}$$

and

$$
S(<_{lin}) = \begin{pmatrix}
2,2,1,0,2,2,1,0,1,2,0,1,0,1,1,0,0,0,1,1,0,2,0,0,2,2,2 \\
2,2,0,1,2,2,1,1,0,1,0,2,0,2,0,2,0,1,0,0,1,1,0,0,1,0,2 \\
1,0,0,1,1,2,2,0,2,2,0,0,0,0,1,0,1,1,0,0,2,2,2,0,0,0,2 \\
0,1,1,1,1,2,1,0,2,1,0,0,0,2,0,1,2,1,0,0,0,2,1,0,1,2,1 \\
2,2,1,1,2,0,0,2,0,1,1,1,0,2,0,2,0,1,0,0,0,1,1,0,0,0,2 \\
2,2,2,2,0,2,0,2,0,0,2,0,0,1,0,1,0,2,0,1,0,2,2,0,0,0,1 \\
1,1,2,1,0,0,0,0,1,1,2,0,2,1,0,1,0,2,0,0,0,1,0,0,2,0,1 \\
0,1,0,0,2,2,0,2,2,2,0,2,2,1,2,1,2,1,0,0,0,2,2,0,1,0,2 \\
1,0,2,2,0,0,1,2,0,1,1,0,1,0,1,2,0,1,0,0,0,1,0,0,0,2,1 \\
2,1,2,1,1,0,1,2,1,1,1,2,0,0,1,2,0,1,1,0,0,0,1,0,2,2,1 \\
0,0,0,0,1,2,2,0,1,1,1,0,0,1,1,2,2,2,0,0,0,2,2,1,0,2,2 \\
1,2,0,0,1,0,0,2,0,2,0,1,0,0,0,0,0,0,0,0,0,0,0,0,0,0,0 \\
0,0,0,0,0,0,2,2,1,0,0,0,1,2,2,2,2,0,0,0,2,2,1,0,1,0,0 \\
1,2,0,2,2,1,1,1,0,0,1,0,2,0,2,0,0,0,0,0,2,0,2,0,2,1,1 \\
1,0,1,0,0,0,0,2,1,1,1,0,2,2,1,2,0,1,0,0,0,0,2,0,2,0,2 \\
0,2,0,1,2,1,1,1,2,2,2,0,2,0,2,0,1,1,0,0,0,0,2,1,2,0,1 \\
0,0,1,2,0,0,0,2,0,0,2,0,2,0,0,1,2,2,0,0,1,0,2,1,2,2,2 \\
0,1,1,1,1,2,2,1,1,1,2,0,0,0,1,1,2,1,0,0,0,0,0,1,0,2,1 \\
1,0,0,0,0,0,0,0,0,1,0,0,0,0,0,0,0,0,1,0,0,0,0,0,0,0,0 \\
1,0,0,0,0,1,0,0,0,0,0,0,0,0,0,0,0,0,1,0,0,0,0,0,0,0,0 \\
0,1,2,0,0,0,0,0,0,0,0,0,2,2,0,0,1,0,0,0,2,1,0,1,2,2,1 \\
2,1,2,2,1,2,1,2,1,0,2,0,2,0,0,0,0,0,0,0,1,1,0,0,0,0,0 \\
0,0,2,1,1,2,0,2,0,1,2,0,1,2,2,2,2,0,0,0,0,0,1,0,0,0,0 \\
0,0,0,0,0,0,0,0,0,0,1,0,0,0,0,1,1,1,0,0,1,0,0,1,0,0,0 \\
2,1,0,1,0,0,2,1,0,2,0,0,1,2,2,2,2,0,0,0,2,0,0,0,1,0,0 \\
2,0,0,2,0,0,0,0,2,2,2,0,0,1,0,0,2,2,0,0,2,0,0,0,0,1,0 \\
2,2,2,1,2,1,1,2,1,1,2,0,0,1,2,1,2,1,0,0,1,0,0,0,0,0,1
\end{pmatrix}
$$

Now, according to the last step of our method, the coordinate vector with respect to the basis $(g_{33\alpha})_{\alpha\in M_3^3(\cdot)}$ *of the output function (the orthogonal solution) is obtained by solving the following system of inhomogeneous linear equations*

$$
\begin{aligned}
A\vec{z} &= \vec{b} \text{ (interpolation conditions)} \\
\vec{y}_i^t S\vec{z} &= 0,\ i=1,...,18 \text{ (orthogonality conditions)}
\end{aligned}
$$

Thus, we obtain

$$\vec{z}(<_{lex}) = (0,0,0,0,0,0,0,0,0,0,0,0,0,0,0,0,2,2,0,0,1,0,0,2,2,0,1)^t$$

and

$$\vec{z}(<_{lin}) = (0,0,0,0,0,0,0,0,0,0,0,2,0,0,0,0,0,0,0,1,0,2,1,2,1,1,1)^t$$

which correspond to the polynomial functions

$$f_{<_{lex}}(\vec{x}) = 2x_1x_3 + 2x_1 + x_2^2 + 2x_2 + 2x_3^2 + 1$$

and

$$f_{<_{lin}}(\vec{x}) = x_1^2x_2^2x_3^2 + x_1^2x_2x_3 + x_1^2x_3^2 + 2x_1^2 + 2x_1x_2^2x_3 + x_1x_2x_3 + x_2^2 + 2x_2x_3^2$$

2 Examples of vector spaces in general position and the codimension condition

Example 2 *Let $n = 2$, $q = 2$ and consider the vector space $F_2(\mathbf{F}_2)$ an its basis $(g_{22\alpha})_{\alpha\in M_2^2} = (x_1x_2, x_1, x_2, 1)$ ordered according to the lexicographic order with $x_1 > x_2$. Furthermore let $U := span(x_1x_2 + x_1 + x_2 + 1)$. The basis vector $u_1 := x_1x_2 + x_1 + x_2 + 1$ has the coordinates $(1, 1, 1, 1)^t$ with respect to the basis $(g_{22\alpha})_{\alpha\in M_2^2}$. Therefore, U is in general position with respect to $(g_{22\alpha})_{\alpha\in M_2^2}$. It is easy to verify*

$$\begin{aligned}|V(U)| &= \left|\{(x,y)\in \mathbf{F}_2^2 \mid xy + x + y + 1 = 0 \mod 2\}\right| \\ &= |\{(0,1),(1,0),(1,1)\}| = 3 \\ &= 2^2 - 1 = codim(U)\end{aligned}$$

As a consequence, the set $X := \{(0,1),(1,0),(1,1)\}$ constitutes an optimal data set to reverse engineer any function $f \in F_2(\mathbf{F}_2)$ displaying no more than 3 terms. If the term-order-free reverse engineering method is used, the probability of successfully retrieving a nonzero function displaying 1 term would be

$$P = \frac{\binom{2^2-1}{2^2-3}}{\binom{2^2}{3}} = \frac{\binom{3}{1}}{\binom{4}{3}} = \frac{3}{4} = 0.75$$

For a function displaying 2 terms $P = 0.5$ and 3 terms $P = 0.25$.

Example 3 *Let $n = 2$, $q = 3$ and consider the vector space $F_2(\mathbf{F}_3)$ an its basis $(g_{23\alpha})_{\alpha\in M_3^2} = (x_1^2x_2^2, x_1^2x_2, x_1x_2^2, x_1^2, x_1x_2, x_2^2, x_1, x_2, 1)$ ordered according to a total degree term order with $x_1 > x_2$. Furthermore let U be the 8-dimensional subspace of $F_2(\mathbf{F}_3)$ generated by*

$$U := span(x_1^2x_2^2 + x_1^2x_2, x_1^2x_2 + x_1x_2^2, x_1x_2^2 + x_1^2, x_1^2 + x_1x_2, x_1x_2 + x_2^2, x_2^2 + x_1, x_1 + x_2, x_2 + 1)$$

The coordinate vectors of the generating vectors are

$$\begin{gathered}\widehat{u}_1 := (1,1,0,...,0)^t \\ \widehat{u}_2 := (0,1,1,0,...,0)^t \\ \vdots \\ \widehat{u}_8 := (0,...,0,1,1)^t\end{gathered}$$

By calculating the determinant of the matrices

$$A_j := \begin{pmatrix}\widehat{u}_1^t \\ \widehat{u}_2^t \\ \vdots \\ \widehat{u}_8^t \\ e_j^t\end{pmatrix},\ j = 1,...,9$$

(where e_j is the jth canonical unit vector of $\mathbf{F}_3^9$), one can easily show that U is in general position with respect to $(g_{23\alpha})_{\alpha\in M_3^2}$. To determine the set $V(U)$, we start solving the three last equations given by

$$\begin{matrix} x_2^2 + x_1 = 0 & & x_2^2 = -1 \\ x_1 + x_2 = 0 & \Leftrightarrow & x_1 = 1 \\ x_2 + 1 = 0 & & x_2 = -1 \end{matrix}$$

This system of equations has no solution in the set $\mathbf{F}_3^2$. Therefore

$$V(U) = \emptyset$$

Consequently, U does not satisfy the codimension condition and thus does not yield an optimal data set.

3 Existence of vector subspaces in general position

The proof is easy but quite technical. The basic idea of the proof is to treat the problem over the real numbers and then construct a solution over finite fields based on the existence of a solution over the real numbers. This last step takes advantage of the density of the rational numbers in the set of real numbers.

We recall the definition of general position for vector spaces over a finite field:

Definition 4 *Let W be a finite dimensional vector space over a finite field $\mathbf{F}_q$ with* $\dim(W) = d > 0$. *Furthermore, let $(w_1, ..., w_d)$ be a fixed basis of W and $s \in \mathbb{N}$ a natural number with $s < d$. A vector subspace $U \subset W$ with* $\dim(U) = s$ *is said to be in* general position *with respect to the basis $(w_1, ..., w_d)$ if for any basis $(v_1, ..., v_s)$ of U and any injective mapping*

$$\pi : \{1, ..., (d-s)\} \to \{1, ..., d\}$$

the vectors

$$v_1, ..., v_s, w_{\pi(1)}, ..., w_{\pi(d-s)} \tag{1}$$

are linearly independent.

It can be easily shown that if the linear independence condition (1) holds for one basis of U, it holds for every other basis of U.

Now we will construct an s-dimensional subspace $U \subset W$ in general position with respect to a given basis of W, where s is an arbitrary natural number with $s < d$. For this purpose we will find the coordinates with respect to the basis $(w_1, ..., w_d)$ of a basis of U. We denote the sought coordinates as follows

$$\vec{\xi}_1 = \begin{pmatrix} x_1 \\ \vdots \\ x_d \end{pmatrix}, \vec{\xi}_2 = \begin{pmatrix} x_{d+1} \\ \vdots \\ x_{2d} \end{pmatrix}, \cdots, \vec{\xi}_s = \begin{pmatrix} x_{(s-1)d+1} \\ \vdots \\ x_{sd} \end{pmatrix}$$

The next step is to count all different injective mappings $\pi : \{1, ..., (d-s)\} \to \{1, ..., d\}$ as $\pi_1, ..., \pi_N$. For each π_i we consider the coordinate vectors $\vec{\xi}_1, ..., \vec{\xi}_s, \vec{e}_{\pi_i(1)}, ..., \vec{e}_{\pi_i(d-s)}$ with respect to the basis $(w_1, ..., w_d)$, where $\vec{e}_j$ is the jth canonical unit vector of $\mathbf{F}_q^d$. Now, for $i = 1, ..., N$ we define the determinant functions

$$\begin{aligned} D_{\pi_i} \quad &: \quad \mathbb{R}^{sd} \to \mathbb{R} \\ \vec{x} \quad &\mapsto \quad \left| \vec{\xi}_1, ..., \vec{\xi}_s, \vec{e}_{\pi_i(1)}, ..., \vec{e}_{\pi_i(d-s)} \right| \end{aligned}$$

where $\vec{e}_j$ is seen as the jth canonical unit vector of $\mathbb{R}^d$. The linear independence condition (1) is equivalent to

$$D_{\pi_i}(\vec{x}) \neq 0$$

Due to the structure of $\left(\vec{\xi}_1, ..., \vec{\xi}_s, \vec{e}_{\pi_i(1)}, ..., \vec{e}_{\pi_i(d-s)}\right)$ and by the Leibniz determinant formula we know that D_{π_i} are nonzero polynomial functions in the variables $x_1, ..., x_{sd}$ and therefore nonzero analytic functions in $\mathbb{R}^{sd}$ with infinite radius of convergence, (in particular, continuous functions). Consequently, no D_{π_i} can be identical to zero on any open subset of $\mathbb{R}^{sd}$. By the continuity of D_{π_1} we know that there is a non-empty open subset $O_1 \subseteq \mathbb{R}^{sd}$ such that $D_{\pi_1}|_{O_1} \neq 0$. Using the same argument we know that there is a non-empty set $O_2 \subseteq O_1$ open in $\mathbb{R}^{sd}$ such that $D_{\pi_2}|_{O_2} \neq 0$. After applying this argument N times we identify a non-empty open subset $O_N \subseteq \mathbb{R}^{sd}$ such that $D_{\pi_i}|_{O_N} \neq 0 \ \forall\ i \in \{1, ..., N\}$. Since the set $\mathbb{Q}^{sd}$ is a dense subset of $\mathbb{R}^{sd}$, there is a point $\vec{y} \in O_N$ with rational entries, i.e. $y_l \in \mathbb{Q}\ \forall\ l \in \{1, ..., sd\}$. Let

$$\vec{y} = (\frac{a_1}{b_1}, ..., \frac{a_{sd}}{b_{sd}})^t$$

and $c := \prod_{k=1}^{sd} b_k$. Since $\vec{y} \in O_N$, we know $D_{\pi_i}(\vec{y}) \neq 0\ \forall\ i \in \{1, ..., N\}$. By the rules of determinants we also know

$$D_{\pi_i}(c\vec{y}) \neq 0\ \forall\ i \in \{1, ..., N\}$$

Moreover, $c\vec{y}$ has integer entries, i.e. $cy_l \in \mathbb{Z}\ \forall\ l \in \{1, ..., sd\}$. For a sufficiently large prime number p, the entries cy_l can be seen as elements of the finite field $\mathbf{F}_p$ of integers modulo p. Therefore, the values $cy_l \in \mathbf{F}_p$, $l = 1, ..., sd$ can be used as the coordinates with respect to the basis $(w_1, ..., w_d)$ of a basis for an s-dimensional subspace $U \subset W$ in general position with respect to the basis $(w_1, ..., w_d)$ of W, a vector space over the finite field $\mathbf{F}_p$. ■

4 The normal form and orthogonality

4.1 Brief Introduction

A well known result by B. Buchberger is the existence of the normal form of a polynomial with respect to a polynomial ideal $I \subseteq K[\tau_1, ..., \tau_n]$ in the ring $K[\tau_1, ..., \tau_n]$ of polynomials in n indeterminates over a field K. This result follows from the existence of so called Gröbner bases for polynomial ideals. For a given fixed term ordering (also known as monomial order), this normal form is unique (Lauer, 1976), (Buchberger, 1976), (Buchberger, 1970). Here we present a new way to calculate this normal form, provided the field K is finite and the ideal I is a vanishing ideal, i.e. I is equal to the set of polynomials which vanish in a given set of points $X \subseteq K^n$. Our method doesn't pursue establishing a new, especially efficient, algorithm for the computation of such a normal form. Rather, the aim of this material is to unveil an interesting way to look at this issue based on the concept of *orthogonality*.

(Sauer, 2001) associated orthogonality with H-bases (introduced by (Macaulay, 1994)) of a polynomial ideal, which are the homogeneous counterpart of Gröbner bases. To this end, (Sauer, 2001) considers inner products on $K[\tau_1, ..., \tau_n]$ (where $K = \mathbb{R}$ or $K = \mathbb{C}$) and calculates the reduced (or normal) form of a polynomial $p \in K[\tau_1, ..., \tau_n]$ with respect to an H-basis using a "nonlinear version" of Gram-Schmidt orthogonalization which *orthogonalizes* leading terms instead of canceling them. One advantage of using H-bases is that they are not tied to term orderings.

In the case that K is a finite field, we were able to associate the concept of orthogonality with the normal form of a polynomial with respect to a Gröbner basis of a vanishing ideal. For orthogonality to apply, we introduce a symmetric bilinear form (see, for instance, (Scharlau, 1969)) on the vector space $K[\tau_1, ..., \tau_n]/I(K^n)$, where $I(K^n) \subset K[\tau_1, ..., \tau_n]$ denotes the vanishing ideal of the set K^n.

In our approach, we consider the orthogonal solution of a solvable inhomogeneous under-determined linear operator equation. If one thinks of an inhomogeneous under-determined system of linear equations in an Euclidean space, the orthogonal solution is simply the solution that is perpendicular to the affine subspace associated with the system. After going through existence and uniqueness considerations, we come to the main statement, namely, that the above mentioned normal form can be obtained as the orthogonal solution of a system of linear equations. That system of equations arises as a linear formulation of the multivariate polynomial interpolation problem.

The concept of orthogonal solution is not tied to monomial orders, as it is the case with Gröbner bases. In this sense, our method reveals a wider class of normal forms (with respect to vanishing ideals) in which the normal forms à la Buchberger appear as special cases.

The organization of the rest of this appendix is the following:

Subsection 4.2 is devoted to the general definition of *symmetric bilinear spaces* and *orthogonal solutions* of an inhomogeneous linear operator equation. Subsection 4.2.1 covers basic definitions and properties of symmetric bilinear spaces. Subsection 4.2.2 introduces the notion of orthogonal solution of a solvable under-determined linear operator equation. Existence and uniqueness of orthogonal solutions are proved and some issues regarding the existence of orthonormal bases are discussed.

Subsection 4.3 deals with the vector space of functions $F : K^n \to K$, where K is a finite field and $n \in \mathbb{N}$. In subsection 4.3.1 we paraphrase the well known result that all the functions in that space are polynomial functions. Subsection 4.3.2 introduces a linear operator called *evaluation epimorphism* and formulates the multivariate polynomial interpolation problem in a linear algebraic fashion.

Subsection 4.4 covers the more technical aspect of constructing special symmetric bilinear forms. Using that type of symmetric bilinear form will allow us to prove the main result of this algebraic study in subsection 4.5.

Subsection 4.5 is devoted to the statement and proof of our main result. Namely, that the canonical normal form of an arbitrary polynomial f with respect to a vanishing ideal $I(X)$ in the ring of multivariate

polynomials over a finite field K can be calculated as the orthogonal solution of a linear operator equation involving the evaluation epimorphism.

For standard terminology, notation and well known results in computational algebraic geometry and commutative algebra we refer to (Cox *et al.* , 1997) and (Becker & Weispfenning, 1993).

4.2 Symmetric bilinear vector spaces and orthogonal solutions of inhomogeneous systems of linear equations

4.2.1 Basic definitions

Let us recall some terminology on symmetric bilinear forms and review some well known basic properties (cf. (Scharlau, 1969)): Let $d \in \mathbb{N}$ and V be a d-dimensional vector space over a field K. A symmetric and bilinear mapping

$$\langle \cdot, \cdot \rangle : V \times V \to K$$

is called *symmetric bilinear form* on V. After fixing a basis $(u_1, ..., u_d)$ of V, it is a well known result, that there is a one-to-one correspondence between the set of all symmetric bilinear forms on V and the set of all $d \times d$ symmetric matrices with entries in K seen as representing matrices with respect to the basis $(u_1, ..., u_d)$. A vector space endowed with a symmetric bilinear form is called a *symmetric bilinear space.* Given a symmetric bilinear space V over a field K, *orthogonality* and *orthonormality* of two vectors $v, w \in V$ as well as the concept of *orthonormal basis* are defined exactly as in the Euclidean case. Similarly, the *orthogonal complement* $W^{\perp} := \{v \in V \mid v \perp w \ \forall\ w \in W\}$ of a subspace $W \subseteq V$ is a subspace of V. Furthermore, if $(w_1, ...w_d)$ is an orthonormal basis of V, then for every vector $v \in V$ holds

$$v = \sum_{k=1}^{d} \langle v, w_k \rangle \, w_k$$

where the field elements $\langle v, w_i \rangle \in K$, $i = 1, ..., d$ are the well known *Fourier coefficients.* Contrary to the case of Euclidean or unitary vector spaces, in symmetric bilinear spaces orthonormal bases don't always exist.

Example 5 *One can construct a symmetric bilinear form on V by setting*

$$\langle u_i, u_j \rangle := \delta_{ij} \ \forall\ i, j \in \{1, ..., d\}$$

Here the basis $(u_1, ...u_d)$ is obviously orthonormal.

4.2.2 Orthogonal solutions of inhomogeneous linear operator equations

Definition 6 *Let $d \in \mathbb{N}$ be a natural number and V a d-dimensional symmetric bilinear space over a field K. Furthermore, let W be an arbitrary vector space over the field K, $T : V \to W$ a non-injective linear operator and $w \in W$ a vector with the property*

$$w \in T(V)$$

Now let $m :=$nullity$(T) \in \mathbb{N}$ be the dimension of the kernel of T. A solution $v^* \in V$ of the equation

$$Tv = w$$

is called orthogonal solution*, if for an arbitrary basis $(u_1, ..., u_m)$ of* $\ker(T)$ *the following orthogonality conditions hold*

$$\langle u_i, v^* \rangle = 0 \ \forall\ i \in \{1, ..., m\}$$

Remark 7 *Let $(u_1, ..., u_m)$ be a basis of* $\ker(T)$*. Then each arbitrary vector $u \in \ker(T)$ can be written in the form*

$$u = \sum_{i=1}^{m} \lambda_i u_i$$

with suitable field elements $\lambda_i \in K$. *If the orthogonality conditions*

$$\langle u_i, v^* \rangle = 0 \ \forall \ i \in \{1, ..., m\}$$

hold for the basis $(u_1, ..., u_m)$, *then we have*

$$\langle u, v^* \rangle = \left\langle \sum_{i=1}^{m} \lambda_i u_i, v^* \right\rangle = \sum_{i=1}^{m} \lambda_i \langle u_i, v^* \rangle = 0$$

and that means

$$v^* \in \ker(T)^{\perp}$$

In particular, for any other different basis $(w_1, ..., w_m)$ *of* $\ker(T)$ *it holds*

$$\langle w_j, v^* \rangle = 0 \ \forall \ j \in \{1, ..., m\}$$

Theorem 8 *Let* $d \in \mathbb{N}$ *be a natural number and* V *a* d*-dimensional symmetric bilinear space over a field* K. *Furthermore, let* W *be an arbitrary vector space over the field* K, $T : V \to W$ *a non-injective linear operator and* $w \in W$ *a vector with the property*

$$w \in T(V)$$

If $\ker(T)$ *has an* orthonormal basis, *then the equation*

$$Tv = w$$

has always a unique orthogonal solution $v^* \in V$.

Proof. Let $m :=$nullity$(T) = \dim(\ker(T)) \in \mathbb{N}$ be the dimension of the null space of T and $(u_1, ..., u_m)$ an orthonormal basis of $\ker(T)$. Since $w \in T(V)$, there must exist a solution $\widehat{\xi} \in V$ of $Tv = w$. For any other solution $\xi \in V$ we have

$$T(\xi - \widehat{\xi}) = T(\xi) - T(\widehat{\xi}) = 0$$

and therefore

$$\xi - \widehat{\xi} \in \ker(T)$$

That means that all solutions $\xi \in V$ of $Tv = w$ can be written in the form

$$\xi = \widehat{\xi} + \sum_{i=1}^{m} \lambda_i u_i$$

with the $\lambda_i \in K$, $i = 1, ..., m$ running over all K. In particular, we can construct a very specific solution by choosing the parameters $\lambda_i \in K$, $i = 1, ..., m$ in the following manner

$$\lambda_i := -\left\langle u_i, \widehat{\xi} \right\rangle, \ i = 1, ..., m$$

For this solution

$$v^* := \widehat{\xi} + \sum_{i=1}^{m} -\left\langle u_i, \widehat{\xi} \right\rangle u_i$$

and for every $j \in \{1, ..., m\}$ it holds

$$\begin{aligned} \langle u_j, v^* \rangle &= \left\langle u_j, \widehat{\xi} + \sum_{i=1}^{m} -\left\langle u_i, \widehat{\xi} \right\rangle u_i \right\rangle = \left\langle u_j, \widehat{\xi} \right\rangle + \sum_{i=1}^{m} -\left\langle u_i, \widehat{\xi} \right\rangle \langle u_j, u_i \rangle \\ &= \left\langle u_j, \widehat{\xi} \right\rangle + \sum_{i=1}^{m} -\left\langle u_i, \widehat{\xi} \right\rangle \delta_{ji} = \left\langle u_j, \widehat{\xi} \right\rangle - \left\langle u_j, \widehat{\xi} \right\rangle = 0 \end{aligned}$$

This shows the existence of an orthogonal solution of $Tv = w$. Now let $\widetilde{v} \in V$ be another orthogonal solution of $Tv = w$. Again, since

$$T(v^* - \widetilde{v}) = T(v^*) - T(\widetilde{v}) = 0$$

we can write

$$v^* = \widetilde{v} + \sum_{i=1}^{m} \alpha_i u_i$$

with suitable $\alpha_i \in K$. From the orthogonality conditions for v^* and $\widetilde{v}$ we have $\forall\ j \in \{1, ..., m\}$

$$\begin{aligned} 0 &= \langle u_i, v^* \rangle = \left\langle u_i, \widetilde{v} + \sum_{i=1}^{m} \alpha_i u_i \right\rangle = \langle u_j, \widetilde{v} \rangle + \left\langle u_j, \sum_{i=1}^{m} \alpha_i u_i \right\rangle \\ &= \sum_{i=1}^{m} \alpha_i \langle u_j, u_i \rangle = \sum_{i=1}^{m} \alpha_i \delta_{ji} = \alpha_j \end{aligned}$$

and that means $v^* = \widetilde{v}$. ∎

Remark 9 *The existence of an orthonormal basis of* $\ker(T)$ *is crucial for the proof of this theorem. It is important to notice that in a symmetric bilinear space over a general field K, the Gram-Schmidt orthonormalization only works if the norm*

$$\|v\| := \sqrt{\langle v, v \rangle}$$

of the vectors used in the Gram-Schmidt process exists in the field K and is not equal to the zero element. In general terms, the existence of square roots would be assured in a field K which satisfies

$$\forall\ x \in K\ \exists\ y \in K \text{ such that } y^2 = x \tag{2}$$

Now, if K is finite, then (2) holds if and only if $Char(K) = 2$.
After fixing a basis $(u_1, ..., u_d)$ for the vector space V, the question whether $\langle v, v \rangle = 0$ for $v \neq 0$ is equivalent to the nontrivial solvability in K^d of the following quadratic form

$$\vec{x}^t A \vec{x} = 0 \tag{3}$$

where A is the representing matrix of $\langle ., . \rangle$ with respect to the basis $(u_1, ..., u_d)$ (see Subsection 4.2.1). In chapter 3, §2 of (Lidl & Niederreiter, 1997) explicit formulas for the exact number of solutions in K^n of equations of the type (3), where A is a $n \times n$ symmetric matrix with entries in a finite field K, can be found.

Corollary 10 *Let K, d, V, W and T be as in the theorem above. If* $\ker(T)$ *has an* orthonormal basis, *then the equation*

$$Tv = 0$$

has always the unique orthogonal solution $0 \in V$.

4.3 The vector space of functions $\mathbf{F}_q^n \to \mathbf{F}_q$

In the next subsection we review the well known result that any function $F : K^n \to K$, where K is a finite field and $n \in \mathbb{N}$, is a polynomial function. Furthermore, we introduce the family of fundamental monomial functions.

4.3.1 The ring of polynomial functions in n variables over $\mathbf{F}_q$ and the vector space of functions $\mathbf{F}_q^n \to \mathbf{F}_q$

Definition 11 *We will denote a finite field with $\mathbf{F}_q$, where q stands for the number of elements of the field (q is a power of the prime characteristic of the field).*

Definition 12 (Notational definition) *We call a commutative Ring $(R, +, \cdot)$ with multiplicative identity $1 \neq 0$ and the binary operations $\cdot$ and $+$ just Ring R.*

The following three results are well known:

Theorem 13 (and Definition) *Let R be a ring and $n \in \mathbb{N}$ a natural number. The set*

$$PF_n(R) := \{g \mid g : R^n \to R \text{ is polynomial}\}$$

together with the common operations $+$ and $\cdot$ of addition and multiplication of mappings is a ring. This ring is called ring of all polynomial functions over R in n R-valued variables.

Theorem 14 (and Definition) *Let K be an arbitrary field and $n \in \mathbb{N}$ a natural number. The set of all functions*

$$f : K^n \to K$$

together with the common operations of addition of mappings and scalar multiplication is a vector space over K. We denote this vector space with $F_n(K)$.

Theorem 15 *Let $\mathbf{F}_q$ be a finite field. Then for the* sets $F_n(\mathbf{F}_q)$ *and* $PF_n(\mathbf{F}_q)$ *it holds*

$$F_n(\mathbf{F}_q) = PF_n(\mathbf{F}_q)$$

Proof. This result is proved in Chapter 7, Section 5 of (Lidl & Niederreiter, 1997). ■

Definition 16 *Let $n, q \in \mathbb{N}$ be natural numbers. Further let $>$ be a total ordering on $(\mathbb{N}_0)^n$. The according to $>$ decreasingly ordered set*

$$M_q^n := \{\alpha \in (\mathbb{N}_0)^n \mid \alpha_j < q \ \forall \ j \in \{1, ..., n\}\}$$

of all n-tuples with entries smaller than q is denoted by $M_q^n \subset (\mathbb{N}_0)^n$.

Remark 17 *In order to avoid a too complicated notation, we skip the appearance of the order relation $>$ in the symbol for this set. It is easy to prove, that M_q^n contains exactly q^n n-tuples. We will index the n-tuples in M_q^n starting with the biggest and ending with the smallest:*

$$\alpha_1 > \alpha_2 > ... > \alpha_{q^n}$$

Definition 18 *For any fixed natural numbers $n, q \in \mathbb{N}$ and for each multi index $\alpha \in M_q^n$ consider the monomial function*

$$\begin{array}{rcl} g_{nq\alpha} & : & K^n \to K \\ \vec{x} & \mapsto & g_{nq\alpha}(\overrightarrow{x}) := \overrightarrow{x}^{\alpha} \end{array}$$

All these monomial functions $g_{nq\alpha}$, $\alpha \in M_q^n$ are called fundamental monomial functions.

The following result is elementary. Its easy induction proof is left to the reader:

Theorem 19 *A basis for the vector space $F_n(\mathbf{F}_q)$ is given by the fundamental monomial functions*

$$(g_{nq\alpha})_{\alpha \in M_q^n}$$

Remark 20 *The basis elements in the basis $(g_{nq\alpha})_{\alpha \in M_q^n}$ are ordered according to the order relation $>$ used to order the n-tuples in the set M_q^n. That means (see Remark 17)*

$$(g_{nq\alpha})_{\alpha \in M_q^n} = (g_{nq\alpha_i})_{i \in \{1, ..., q^n\}}$$

4.3.2 Solving the polynomial interpolation problem in $PF_n(\mathbf{F}_q)$

In this subsection we define the *evaluation epimorphism* of a tuple $(\vec{x}_1, ..., \vec{x}_m) \in (\mathbf{F}_q^n)^m$ of points in the space $\mathbf{F}_q^n$. The evaluation epimorphism allows for a linear algebraic formulation of the multivariate polynomial interpolation problem.

Theorem 21 (and Definition) *Let $\mathbf{F}_q$ be a finite field and $n, m \in \mathbb{N}$ natural numbers with $m \leq q^n$. Further let*

$$\vec{X} := (\vec{x}_1, ..., \vec{x}_m) \in (\mathbf{F}_q^n)^m$$

*be a tuple of m **different** n-tuples with entries in the field $\mathbf{F}_q$. Then the mapping*

$$\begin{array}{rcl} \Phi_{\vec{X}} & : & F_n(\mathbf{F}_q) \to \mathbf{F}_q^m \\ f & \mapsto & \Phi_{\vec{X}}(f) := (f(\vec{x}_1), ..., f(\vec{x}_m))^t \end{array}$$

is a surjective linear operator. $\Phi_{\vec{X}}$ is called the evaluation epimorphism of the tuple $\vec{X}$.

Proof. The proof of the linearity is left to the reader. Now let $\vec{b} \in \mathbf{F}_q^m$ be an arbitrary vector. Since $m \leq q^n$ we can construct a function

$$g \in F_n(\mathbf{F}_q)$$

with the property

$$g(\vec{x}_i) = b_i \;\forall\; i \in \{1, ..., m\}$$

and that means exactly

$$\Phi_{\vec{X}}(g) = \vec{b} \quad \blacksquare$$

Definition 22 (Notational Definition) *Let be $n\,, m \in \mathbb{N}$ natural numbers and K a field. The set of all $m \times n$ matrices (m* rows and n columns*) with entries in K is denoted by $M(m \times n;\, K)$.*

Remark 23 (and Corollary) *Since a basis of $F_n(\mathbf{F}_q)$ is given by the fundamental monomial functions $(g_{nq\alpha})_{\alpha \in M_q^n}$, the matrix*

$$A := (\Phi_{\vec{X}}(g_{nq\alpha}))_{\alpha \in M_q^n} \in M(m \times q^n; \mathbf{F}_q)$$

representing the evaluation epimorphism $\Phi_{\vec{X}}$ of the tuple $\vec{X}$ with respect to the basis $(g_{nq\alpha})_{\alpha \in M_q^n}$ of $F_n(\mathbf{F}_q)$ and the canonical basis of $\mathbf{F}_q^m$ has always the full rank $m = \min(m, q^n)$. That also means, that the dimension of the $\ker(\Phi_{\vec{X}})$ *is*

$$\dim(\ker(\Phi_{\vec{X}})) = \dim(F_n(\mathbf{F}_q)) - m = q^n - m$$

Corollary 24 *Let $\mathbf{F}_q$ be a finite field and $n, m \in \mathbb{N}$ natural numbers with $m \leq q^n$. Further let*

$$\vec{X} := (\vec{x}_1, ..., \vec{x}_m) \in (\mathbf{F}_q^n)^m$$

be a tuple of m different n-tuples with entries in the field $\mathbf{F}_q$ and $\vec{b} \in \mathbf{F}_q^m$ a vector. Then the interpolation problem of finding a polynomial function $f \in PF_n(\mathbf{F}_q)$ with the property

$$f(\vec{x}_i) = b_i \;\forall\; i \in \{1, ..., m\}$$

can be solved by solving the system of linear equations

$$A\vec{y} = \vec{b} \tag{4}$$

where

$$A := (\Phi_{\vec{X}}(g_{nq\alpha}))_{\alpha \in M_q^n}$$

is the matrix representing the evaluation epimorphism $\Phi_{\vec{X}}$ of the tuple $\vec{X}$ with respect to the basis $(g_{nq\alpha})_{\alpha \in M_q^n}$ of $F_n(\mathbf{F}_q)$ and the canonical basis of $\mathbf{F}_q^m$. The entries of a solution vector of the equations (4) are the coefficients of the solution with respect to the basis $(g_{nq\alpha})_{\alpha \in M_q^n}$.

4.4 Construction of special purpose symmetric bilinear forms

Let $\mathbf{F}_q$ be a finite field and $n, m \in \mathbb{N}$ natural numbers with $m < q^n$. Further let

$$\vec{X} := (\vec{x}_1, ..., \vec{x}_m) \in \left(\mathbf{F}_q^n\right)^m$$

be a tuple of m different n-tuples with entries in the field $\mathbf{F}_q$ and $d := \dim(F_n(\mathbf{F}_q))$. Now consider the evaluation epimorphism $\Phi_{\vec{X}}$ of the tuple $\vec{X}$. By Remark 23 and due to the fact $m < q^n$, the nullity of $\Phi_{\vec{X}}$ is given by

$$s := \dim(\ker(\Phi_{\vec{X}})) = \dim(F_n(\mathbf{F}_q)) - m = q^n - m > 0$$

Now let $(u_1, ..., u_s)$ be a basis of $\ker(\Phi_{\vec{X}}) \subseteq F_n(\mathbf{F}_q)$. By the basis extension theorem, we can extend the basis $(u_1, ..., u_s)$ to a basis

$$(u_1, ..., u_s, u_{s+1}, ..., u_d)$$

of the whole space $F_n(\mathbf{F}_q)$. As stated in Subsection 4.2.1, we can construct a symmetric bilinear form on $F_n(\mathbf{F}_q)$ by setting

$$\langle u_i, u_j \rangle := \delta_{ij} \ \forall \ i, j \in \{1, ..., d\}$$

Here the basis $(u_1, ...u_d)$ is orthonormal and the vectors $(u_{s+1}, ..., u_d)$ are a basis of the orthogonal complement $\ker(\Phi_{\vec{X}})^\perp$ of $\ker(\Phi_{\vec{X}})$.

In general, the way we extend the basis $(u_1, ..., u_s)$ of $\ker(\Phi_{\vec{X}})$ to a basis

$$(u_1, ..., u_s, u_{s+1}, ..., u_d)$$

of the whole space $F_n(\mathbf{F}_q)$ determines crucially the symmetric bilinear form we get by setting $\langle u_i, u_j \rangle := \delta_{ij}$ $\forall \ i, j \in \{1, ..., d\}$. Consequently, the orthogonal solution of $\Phi_{\vec{X}}(g) = \vec{b}$ may vary according to the chosen extension $u_{s+1}, ..., u_d \in F_n(\mathbf{F}_q)$. One systematic way to get a basis of the whole space $F_n(\mathbf{F}_q)$ starting with a basis $(u_1, ..., u_s)$ of $\ker(\Phi_{\vec{X}})$ is the following: let

$$(\vec{y}_1, ..., \vec{y}_s)^t \tag{5}$$

be the matrix whose rows are the coordinate vectors $\vec{y}_1, ..., \vec{y}_s \in K^d$ of $(u_1, ..., u_s)$ with respect to the basis $(g_{nq\alpha})_{\alpha \in M_q^n}$ of $F_n(\mathbf{F}_q)$. Now we perform Gauss-Jordan elimination on the matrix (5), obtaining the matrix R. Now consider the set $B := \{\vec{e}_1, ..., \vec{e}_d\}$ of canonical unit vectors of the space $\mathbf{F}_q^d$. For every pivot element r_{ij} used during the Gauss-Jordan elimination performed on (5), eliminate the canonical unit vector $\vec{e}_j$ from the set B. This yields the set $\tilde{B}$. The coordinate vectors for a basis for the whole space $F_n(\mathbf{F}_q)$ are now given by the the rows of R and the vectors in the set $\tilde{B}$. We call this way of construction of the orthonormal basis for the space $F_n(\mathbf{F}_q)$ the *standard orthonormalization.* We illustrate the algorithm using an example:

Example 25 *Suppose $q = 3$, $\mathbf{F}_3 = \mathbb{Z}_3$, $m = 4$, $d = 3^2 = 9$, $s = 5$ and that after performing Gauss-Jordan elimination on (5) we get the following matrix*

$$R := \begin{pmatrix} 1 & 0 & z_{1,3} & 0 & 0 & z_{1,6} & 0 & z_{1,8} & z_{1,9} \\ 0 & 1 & z_{2,3} & 0 & 0 & z_{2,6} & 0 & z_{2,8} & z_{2,9} \\ 0 & 0 & 0 & 1 & 0 & z_{3,6} & 0 & z_{3,8} & z_{3,9} \\ 0 & 0 & 0 & 0 & 1 & z_{4,6} & 0 & z_{4,8} & z_{4,9} \\ 0 & 0 & 0 & 0 & 0 & 0 & 1 & z_{5,8} & z_{5,9} \end{pmatrix} \tag{6}$$

(The $z_{i,j} \in \mathbf{F}_q$ stand for unspecified field elements). Then for the extension of the basis we choose the following canonical basis vectors

$$\vec{e}_3, \vec{e}_6, \vec{e}_8, \vec{e}_9 \in \mathbb{Z}_3^9$$

Now we substitute coordinate vectors $(\vec{y}_1, ..., \vec{y}_5)$ of the basis $(u_1, ..., u_5)$ by the rows in the reduced matrix 6 (this step is not strictly necessary, but it will be needed to prove the theorems below) and get the following coordinate vectors for a basis for the whole space $F_2(\mathbb{Z}_3)$

$$(\widetilde{\vec{y}_1}, ..., \widetilde{\vec{y}_s}, \vec{y}_{s+1}, ..., \vec{y}_d) := \left(R^t, \vec{e}_3, \vec{e}_6, \vec{e}_8, \vec{e}_9\right)$$

In this specific example we use the standard lexicographic ordering on $(\mathbb{N}_0)^2$ *and so we have*

$$M_3^2 = \{(2,2),(2,1),(2,0),(1,2),(1,1),(1,0),(0,2),(0,1),(0,0)\}$$

and

$$(g_{23\alpha}(\vec{x}))_{\alpha \in M_3^2} = \left(x_2^2x_1^2, x_2^2x_1, x_2^2, x_2x_1^2, x_2x_1, x_2, x_1^2, x_1, 1\right)$$

Thus the orthonormal basis $(\widetilde{u_1}, ..., \widetilde{u_s}, u_{s+1}, ..., u_d)$ *of* $F_2(\mathbb{Z}_3)$ *evaluated at the point* $\vec{x} \in \mathbb{Z}_3^2$ *would be*

$$\begin{pmatrix} x_2^2x_1^2 + z_{1,3}x_2^2 + z_{1,6}x_2 + z_{1,8}x_1 + z_{1,9} \\ x_2x_1^2 + z_{2,3}x_2^2 + z_{2,6}x_2 + z_{2,8}x_1 + z_{2,9} \\ x_2x_1^2 + z_{3,6}x_2 + z_{3,8}x_1 + z_{3,9} \\ x_2x_1 + z_{4,6}x_2 + z_{4,8}x_1 + z_{4,9} \\ x_1^2 + z_{5,8}x_1 + z_{5,9} \\ x_2^2 \\ x_2 \\ x_1 \\ 1 \end{pmatrix}^t$$

and the orthogonal solution of $\Phi_{\vec{X}}(g) = \vec{b}$ *is a vector in Span*$(x_2^2\ ,\ x_2\ ,\ x_1\ ,\ 1)$.

In the next subsection, we will establish the exact relationship between the orthogonal solution of $\Phi_{\vec{X}}(g) = \vec{b}$ (using the symmetric bilinear form defined above) and the normal form with respect to the vanishing ideal $I(X)$. This relationship can be established if the order relation $>$ used to order the n-tuples in the set M_q^n is a *monomial ordering.* If, more generally, total orderings on $(\mathbb{N}_0)^n$ are used to order the set M_q^n, the set of possible orthogonal solutions of $\Phi_{\vec{X}}(g) = \vec{b}$ can be seen as a wider class of normal forms (with respect to vanishing ideals) in which the "classical" normal forms (attached to monomial orderings) appear as special cases.

4.5 Orthogonal solutions of $\Phi_{\vec{X}}(g) = \vec{b}$ and the normal form with respect to the vanishing ideal $I(X)$

In this subsection we will show the main result of this appendix: Given a set of points $X \subset K^n$, an arbitrary polynomial $f \in K[\tau_1, ..., \tau_n]$ and a monomial order $>$, the normal form of f with respect to the vanishing ideal $I(X) \subseteq K[\tau_1, ..., \tau_n]$ can be calculated as the orthogonal solution of

$$\Phi_{\vec{X}}(g) = \vec{b}$$

where $\vec{b}$ is given by

$$b_i := \widetilde{f}(\vec{x}_i),\ i = 1, ..., m$$

The yet undefined notation $\widetilde{f}$ suggests that a mapping between the ring $K[\tau_1, ..., \tau_n]$ of polynomials and the vector space of functions $F_n(\mathbf{F}_q)$ is needed. That mapping will be defined and characterized in the first lemma and theorem of this subsection. After introducing some notation we arrive at an important preliminary result in Theorem 30, which states how a (particular) basis of $\ker(\Phi_{\vec{X}})$ can be extended to a Gröbner basis of $I(X)$. With that result our goal can be easily reached. Please note that through this subsection a more technical result stated and proved in the Technical Results Subsection (see below) is used.

Lemma 26 (and Definition) *Let* K *be a field,* $n, q \in \mathbb{N}$ *natural numbers and* $K[\tau_1, ..., \tau_n]$ *the polynomial ring in n indeterminates over* K*. Then the set of all polynomials of the form*

$$\sum_{\alpha \in M_q^n} a_\alpha \tau_1^{\alpha_1} ... \tau_n^{\alpha_n} \in K[\tau_1, ..., \tau_n]$$

with coefficients $a_\alpha \in K$ *is a vector space over* K*. We denote this set with* $P_q^n(K) \subset K[\tau_1, ..., \tau_n]$.

Proof. The easy proof is left to the reader. ■

Theorem 27 *Let $\mathbf{F}_q$ be a finite field and $n \in \mathbb{N}$ a natural number. Then the vector spaces $P_q^n(\mathbf{F}_q)$ and $F_n(\mathbf{F}_q)$ are isomorphic.*

Proof. After defining the linear mapping

$$\begin{aligned} \varphi &: P_q^n(\mathbf{F}_q) \to F_n(\mathbf{F}_q) \\ g &= \sum_{\alpha \in M_q^n} a_\alpha \tau_1^{\alpha_1} ... \tau_n^{\alpha_n} \mapsto \varphi(g)(\vec{x}) := \sum_{\alpha \in M_q^n} a_\alpha \overrightarrow{x}^\alpha \end{aligned}$$

the claim follows easily. ■

Remark 28 (and Definition) *The mapping φ is defined on the set $P_q^n(K) \subset K[\tau_1, ..., \tau_n]$, but of course it can naturally be extended to $K[\tau_1, ..., \tau_n]$ as*

$$\begin{aligned} \varphi &: K[\tau_1, ..., \tau_n] \to F_n(\mathbf{F}_q) \\ g &= \sum_{\alpha \in \Gamma} a_\alpha \tau_1^{\alpha_1} ... \tau_n^{\alpha_n} \mapsto \varphi(g)(\vec{x}) := \sum_{\alpha \in \Gamma} a_\alpha \overrightarrow{x}^\alpha \end{aligned}$$

where Γ is a finite set of multi indexes. We denote the image under $\varphi : K[\tau_1, ..., \tau_n] \to F_n(\mathbf{F}_q)$ of a polynomial $g \in K[\tau_1, ..., \tau_n]$ with

$$\widetilde{g} := \varphi(g) \in F_n(\mathbf{F}_q)$$

Definition 29 *Let $d \in \mathbb{N}$ be a natural number, V a d-dimensional vector space over a field K and F a basis of V. Furthermore, let $U \subset V$ be an arbitrary* proper *subspace of V. Now let $s := \dim(U) \in \mathbb{N}$. A basis $(u_1, ..., u_s)$ of U is called a* cleaned kernel basis with respect to the basis F *if the matrix $(\vec{y}_1, ..., \vec{y}_s)^t$ whose rows are the coordinate vectors $\vec{y}_1, ..., \vec{y}_s \in K^d$ of $(u_1, ..., u_s)$ with respect to the basis F is in reduced row echelon form.*

For a tuple $\vec{x} = (x_1, ..., x_n)$ we write $x := \{x_1, ..., x_n\}$ for the set containing all the entries in the tuple $\vec{x}$.

Theorem 30 *Let $\mathbf{F}_q$ be a finite field, $n, m \in \mathbb{N}$ natural numbers with $m < q^n$ and $>$ a fixed monomial order. Further let*

$$\vec{X} := (\vec{x}_1, ..., \vec{x}_m) \in \left(\mathbf{F}_q^n\right)^m$$

be a tuple of m different n-tuples with entries in the field $\mathbf{F}_q$ and $s := \dim(\ker(\Phi_{\vec{X}}))$. In addition, let $(u_1, ..., u_s)$ be a cleaned kernel basis of $\ker(\Phi_{\vec{X}}) \subseteq F_n(\mathbf{F}_q)$ with respect to the basis $(g_{nq\alpha})_{\alpha \in M_q^n}$. Then the family of polynomials

$$\left(\tau_1^q - \tau_1, \tau_2^q - \tau_2, ..., \tau_n^q - \tau_n, \varphi^{-1}(u_1), ..., \varphi^{-1}(u_s)\right)$$

is a Gröbner basis of the vanishing ideal $I(X) \subseteq \mathbf{F}_q[\tau_1, ..., \tau_n]$ with respect to the monomial order $>$.

Proof. The idea of the proof is to show that

$$U := \left(\tau_1^q - \tau_1, \tau_2^q - \tau_2, ..., \tau_n^q - \tau_n, \varphi^{-1}(u_1), ..., \varphi^{-1}(u_s)\right)$$

generates the ideal $I(X)$ and that for any polynomial $g \in I(X)$ the remainder on division of g by U is zero. According to a well known fact about Gröbner bases (see proposition 5.38 of (Becker & Weispfenning, 1993)) this is equivalent to U being a Gröbner basis for $I(X)$. For this proof, remember that the fundamental monomial functions $(g_{nq\alpha})_{\alpha \in M_q^n}$ are ordered decreasingly with respect to the order $>$.
Now let $g \in I(X) \subseteq \mathbf{F}_q[\tau_1, ..., \tau_n]$ be an arbitrary polynomial in the vanishing ideal of X. Since

$$(\tau_1^q - \tau_1, \tau_2^q - \tau_2, ..., \tau_n^q - \tau_n)$$

is a universal Gröbner basis for $I(\mathbf{F}_q^n)$ (see Theorem 36 in the appendix), there is a unique $r \in \mathbf{F}_q[\tau_1, ..., \tau_n]$ with the properties

1. No term of r is divisible by any of $LT(\tau_1^q - \tau_1) = \tau_1^q, LT(\tau_2^q - \tau_2) = \tau_2^q, ..., LT(\tau_n^q - \tau_n) = \tau_n^q$. That means in particular $r \in P_q^n(\mathbf{F}_q)$.
2. There is a $q \in I(\mathbf{F}_q^n)$ such that $g = q + r$

This means that when we start to divide g by the (ordered) family U we get the intermediate result

$$g = q + r$$

where the remainder $r \in P_q^n(\mathbf{F}_q)$ and $q \in \langle \tau_1^q - \tau_1, \tau_2^q - \tau_2, ..., \tau_n^q - \tau_n \rangle = I(\mathbf{F}_q^n)$. If $r = 0$, then we are done and the remainder $\bar{g}^U$ on division of g by U is zero. If $r \neq 0$, then we know from

$$r = g - q$$

that $r \in I(X)$ ($q \in I(\mathbf{F}_q^n) \subseteq I(X)$) and this is equivalent to

$$\widetilde{r}(\vec{x}) = \varphi(r)(\vec{x}) = 0 \ \forall \ \vec{x} \in \mathbf{F}_q^n \Leftrightarrow \widetilde{r} \in \ker(\Phi_{\vec{X}})$$

Since $(u_1, ..., u_s)$ is a basis for $\ker(\Phi_{\vec{X}})$, there are unique $\lambda_i \in \mathbf{F}_q$, $i = 1, ..., s$ with

$$\widetilde{r} = \sum_{i=1}^{s} \lambda_i u_i$$

Applying the vector space isomorphism $\varphi^{-1} : F_n(\mathbf{F}_q) \to P_q^n(\mathbf{F}_q)$ to this equation yields

$$r = \sum_{i=1}^{s} \lambda_i \varphi^{-1}(u_i)$$

From the requirement on $(u_1, ..., u_s)$ to be a cleaned kernel basis of $\ker(\Phi_{\vec{X}})$ now follows for each $j \in \{1, ..., s\}$, that the leading term

$$LT(\varphi^{-1}(u_j))$$

doesn't appear in the polynomials $\varphi^{-1}(u_i)$, $i \in \{1, ..., s\} \backslash \{j\}$. Consequently, in the expression

$$\sum_{i=1}^{s} \lambda_i \varphi^{-1}(u_i)$$

no cancellation of the leading terms $LT(\varphi^{-1}(u_i))$, $i = 1, ..., s$ can occur. Therefore, the division of $r = \sum_{i=1}^{s} \lambda_i \varphi^{-1}(u_i)$ by $\big(\varphi^{-1}(u_1), ..., \varphi^{-1}(u_s)\big)$ must yield

$$r = \sum_{i=1}^{s} \lambda_i \varphi^{-1}(u_i) + 0$$

and the remainder $\bar{g}^U$ on division of g by U is zero. As a consequence,

$$g \in \big\langle \tau_1^q - \tau_1, \tau_2^q - \tau_2, ..., \tau_n^q - \tau_n, \varphi^{-1}(u_1), ..., \varphi^{-1}(u_s) \big\rangle$$

and since $g \in I(X)$ was arbitrary

$$I(X) \subseteq \big\langle \tau_1^q - \tau_1, \tau_2^q - \tau_2, ..., \tau_n^q - \tau_n, \varphi^{-1}(u_1), ..., \varphi^{-1}(u_s) \big\rangle$$

The inclusion

$$\big\langle \tau_1^q - \tau_1, \tau_2^q - \tau_2, ..., \tau_n^q - \tau_n, \varphi^{-1}(u_1), ..., \varphi^{-1}(u_s) \big\rangle \subseteq I(X)$$

is given by the fact $u_1, ..., u_s \in \ker(\Phi_{\vec{X}})$ and Theorem 36. Summarizing we can say

$$\big\langle \tau_1^q - \tau_1, \tau_2^q - \tau_2, ..., \tau_n^q - \tau_n, \varphi^{-1}(u_1), ..., \varphi^{-1}(u_s) \big\rangle = I(X)$$

and for every $g \in I(X)$ the remainder $\bar{g}^U$ on division of g by U is zero. Now proposition 5.38 of (Becker & Weispfenning, 1993) (see also the remarks after corollary 2, chapter 2, § 6 of (Cox *et al.* , 1997)) proves the claim. ■

Theorem 31 *Let $\mathbf{F}_q$ be a finite field, $n, m \in \mathbb{N}$ natural numbers with $m < q^n$ and $>$ a fixed monomial order. Further let*

$$\vec{X} := (\vec{x}_1, ..., \vec{x}_m) \in (\mathbf{F}_q^n)^m$$

be a tuple of m different n-tuples with entries in the field $\mathbf{F}_q$, $\vec{b} \in \mathbf{F}_q^m$ a vector, $d := \dim(F_n(\mathbf{F}_q))$ and $s := \dim(\ker(\Phi_{\vec{X}}))$. In addition, let $(u_1, ..., u_s)$ be a cleaned *kernel basis of* $\ker(\Phi_{\vec{X}}) \subseteq F_n(\mathbf{F}_q)$ *with respect to the basis $(g_{nq\alpha})_{\alpha \in M_q^n}$, $(u_1, ..., u_s, u_{s+1}, ..., u_d)$ an orthonormal basis of $F_n(\mathbf{F}_q)$ constructed* using the standard orthonormalization *and $f \in \mathbf{F}_q[\tau_1, ..., \tau_n]$ a polynomial satisfying the interpolation conditions*

$$\widetilde{f}(\vec{x}_j) = b_j \ \forall \ j \in \{1, ..., m\}$$

Furthermore, let $U \subseteq I(X)$ be an arbitrary Gröbner basis of the vanishing ideal $I(X)$ with respect to the monomial order $>$ and v^* the orthogonal solution of $\Phi_{\vec{X}}(g) = \vec{b}$. Then

$$\varphi^{-1}(v^*) = \overline{f}^U$$

Proof. If $\varphi^{-1}(v^*) = 0$ then $v^* = 0$ and

$$\vec{b} = \Phi_{\vec{X}}(v^*) = \Phi_{\vec{X}}(0) = \vec{0}$$

In this case we also have

$$\overline{f}^U = 0$$

and therefore

$$\varphi^{-1}(v^*) = \overline{f}^U$$

Assume $\varphi^{-1}(v^*) \neq 0$. Since the remainder on division by a Gröbner basis is independent of which Gröbner basis we use (for a fixed monomial order), the idea of the proof is to show that $\varphi^{-1}(v^*)$ is the unique remainder on division by the Gröbner basis

$$\left(\tau_1^q - \tau_1, \tau_2^q - \tau_2, ..., \tau_n^q - \tau_n, \varphi^{-1}(u_1), ..., \varphi^{-1}(u_s)\right)$$

(see Theorem 30). Now, since $\varphi^{-1}(v^*) \in P_q^n(\mathbf{F}_q)$, no term of $\varphi^{-1}(v^*)$ is divisible by any of the

$$LT(\tau_1^q - \tau_1) = \tau_1^q, LT(\tau_2^q - \tau_2) = \tau_2^q, ..., LT(\tau_n^q - \tau_n) = \tau_n^q$$

If terms of $\varphi^{-1}(v^*)$ would be divisible by

$$LT(\varphi^{-1}(u_1)), ..., LT(\varphi^{-1}(u_s))$$

then after division by the family

$$\left(\tau_1^q - \tau_1, \tau_2^q - \tau_2, ..., \tau_n^q - \tau_n, \varphi^{-1}(u_1), ..., \varphi^{-1}(u_s)\right)$$

we would have

$$\varphi^{-1}(v^*) = \sum_{i=1}^{s} h_i \varphi^{-1}(u_i) + r \tag{7}$$

where $h_i, r \in \mathbf{F}_q[\tau_1, ..., \tau_n]$, $i = 1, ..., s$ and either $r = 0$ or no term of r is divisible by the

$$LT(\tau_1^q - \tau_1), ..., LT(\tau_n^q - \tau_n), LT(\varphi^{-1}(u_1)), ..., LT(\varphi^{-1}(u_s))$$

If $r = 0$, then

$$\varphi^{-1}(v^*) = \sum_{i=1}^{s} h_i \varphi^{-1}(u_i)$$

and the polynomial $\varphi^{-1}(v^*)$ vanishes on the set X, that is

$$\varphi(\varphi^{-1}(v^*))(\vec{x}) = v^*(\vec{x}) = 0 \ \forall \ \vec{x} \in X$$

Consequently

$$\vec{b} = \Phi_{\vec{X}}(v^*) = \vec{0}$$

and due to the uniqueness of the orthogonal solution

$$v^* = 0$$

But this is a contradiction to our assumption $\varphi^{-1}(v^*) \neq 0$.
Now if $r \neq 0$, since no term of r is divisible by $LT(\tau_1^q - \tau_1), ..., LT(\tau_n^q - \tau_n)$, then in particular $r \in P_q^n(\mathbf{F}_q)$. Due to the fact, that $(u_1, ..., u_s, u_{s+1}, ..., u_d)$ is a basis for $F_n(\mathbf{F}_q)$, we can write

$$\widetilde{r} = \varphi(r) = \sum_{j=1}^{d} \lambda_j u_j$$

with unique $\lambda_j \in \mathbf{F}_q$, $j = 1, ..., d$. Applying the vector space isomorphism $\varphi^{-1} : F_n(\mathbf{F}_q) \to P_q^n(\mathbf{F}_q)$ to this equation yields

$$r = \sum_{j=1}^{d} \lambda_j \varphi^{-1}(u_j)$$

From the requirement on $(u_1, ..., u_s)$ to be a cleaned kernel basis of $\ker(\Phi_{\vec{X}})$ with respect to the basis $(g_{nq\alpha})_{\alpha \in M_q^n}$ and since the basis extension $(u_1, ..., u_s, u_{s+1}, ..., u_d)$ has been constructed using the standard orthonormalization, in the expression

$$\sum_{j=1}^{d} \lambda_j \varphi^{-1}(u_j)$$

no cancellation of the leading terms $LT(\varphi^{-1}(u_k))$, $k = 1, ..., s$ can occur. But r is not divisible by $LT(\varphi^{-1}(u_1)), ..., LT(\varphi^{-1}(u_s))$ and that forces

$$\lambda_k = 0, \ \forall \ k \in \{1, ..., s\}$$

In other words

$$r = \sum_{j=s+1}^{d} \lambda_j \varphi^{-1}(u_j) \Leftrightarrow \widetilde{r} = \varphi(r) = \sum_{j=s+1}^{d} \lambda_j u_j$$

which is equivalent to

$$\widetilde{r} \in \ker(\Phi_{\vec{X}})^{\perp} \tag{8}$$

From the equation (7) we know that

$$r = \varphi^{-1}(v^*) - \sum_{i=1}^{s} h_i \varphi^{-1}(u_i)$$

and that means

$$\widetilde{r}(\vec{x}) = v^*(\vec{x}) \ \forall \ \vec{x} \in X$$

In other words

$$\Phi_{\vec{X}}(\widetilde{r}) = \vec{b}$$

This together with (8) says that $\widetilde{r}$ is an orthogonal solution of $\Phi_{\vec{X}}(g) = \vec{b}$. From the uniqueness now follows

$$v^* = \widetilde{r} \Leftrightarrow \varphi^{-1}(v^*) = r$$

Consequently, no term of the polynomial $\varphi^{-1}(v^*)$ is divisible by any of the leading terms of the elements of the Gröbner basis (see Theorem 30)

$$G := \left(\tau_1^q - \tau_1, \tau_2^q - \tau_2, ..., \tau_n^q - \tau_n, \varphi^{-1}(u_1), ..., \varphi^{-1}(u_s)\right)$$

for the vanishing ideal $I(X)$. Now we define the polynomial

$$h := f - \varphi^{-1}(v^*)$$

Since v^* is a solution of $\Phi_{\vec{X}}(g) = \vec{b}$ and f satisfies the interpolation conditions

$$\widetilde{f}(\vec{x}_j) = b_j \;\forall\; j \in \{1, ..., m\}$$

we have

$$\widetilde{h}(\vec{x}) = \widetilde{f}(\vec{x}) - v^*(\vec{x}) = 0 \;\forall\; \vec{x} \in X \Leftrightarrow h \in I(X)$$

So we have a polynomial $h \in I(X)$ such that

$$f = h + \varphi^{-1}(v^*)$$

By proposition 1, chapter 2, §6 in (Cox *et al.* , 1997), $\varphi^{-1}(v^*)$ is the unique remainder on division by the Gröbner basis G. It is a well known fact, that the remainder on division by a Gröbner basis is independent of which Gröbner basis we use, as long as we use one fixed particular monomial order. Therefore

$$\overline{f}^U = \overline{f}^G = \varphi^{-1}(v^*) \quad \blacksquare$$

Remark 32 (and main theorem) *Let $\mathbf{F}_q$ be a finite field, $n, m \in \mathbb{N}$ natural numbers with $m < q^n$ and $>$ a fixed monomial order. Further let*

$$\vec{X} := (\vec{x}_1, ..., \vec{x}_m) \in (\mathbf{F}_q^n)^m$$

be a tuple of m different n-tuples with entries in the field $\mathbf{F}_q$, $U \subseteq I(X)$ an arbitrary Gröbner basis of the vanishing ideal $I(X)$ and $f \in \mathbf{F}_q[\tau_1, ..., \tau_n]$ an arbitrary *polynomial. Then*

$$\overline{f}^U = \varphi^{-1}(v^*)$$

where v^* is the orthogonal solution of $\Phi_{\vec{X}}(g) = \vec{b}$ and $\vec{b}$ is given by

$$b_i := \widetilde{f}(\vec{x}_i), \;\; i = 1, ..., m$$

Remark 33 *Let*

$$A := (\Phi_{\vec{X}}(g_{nq\alpha}))_{\alpha \in M_q^n} \in M(m \times q^n; \mathbf{F}_q)$$

be the matrix representing the evaluation epimorphism $\Phi_{\vec{X}}$ of the tuple $\vec{X}$ with respect to the basis $(g_{nq\alpha})_{\alpha \in M_q^n}$ of $F_n(\mathbf{F}_q)$ and the canonical basis of $\mathbf{F}_q^m$ and S the matrix

$$S_{ij} := \left\langle g_{nq\alpha_i}, g_{nq\alpha_j} \right\rangle, \;\; i, j \in \{1, ..., q^n\}$$

representing the symmetric bilinear form with respect to the basis $(g_{nq\alpha})_{\alpha \in M_q^n}$. Further let $\vec{y}_1, ..., \vec{y}_s \in \mathbf{F}_q^d$ be the coordinate vectors of $(u_1, ..., u_s)$ with respect to the basis $(g_{nq\alpha})_{\alpha \in M_q^n}$. Then the above result states that the normal form $\overline{f}^U$ of f with respect to the Gröbner basis $U \subseteq I(X)$ can be calculated by solving the following system of inhomogeneous linear equations

$$\begin{aligned} A\vec{z} &= \vec{b} \\ \vec{y}_i^t S \vec{z} &= 0, \;\; i = 1, ..., s \end{aligned}$$

4.6 Acknowledgements

We would like to thank Dr. Gretchen Matthews, Dr. Michael Shapiro and Dr. Michael Stillman for very helpful comments and contributions for the material presented in this appendix. The author acknowledges support by a Public Health Service grant (RO1 AI062989) to David Thorley-Lawson at Tufts University, Boston, MA.

4.7 Technical Results

Lemma 34 *Let K be a field, $n \in \mathbb{N}$ a natural number, $K[\tau_1, ..., \tau_n]$ the polynomial ring in n indeterminates over K and $>$ an arbitrary monomial order. Then for each natural number $m \in \mathbb{N}$ and each $i \in \{1, ..., n\}$ it holds*

$$\tau_i^m > \tau_i^{m-1} > ... > \tau_i > \tau_i^0 \tag{9}$$

Proof. The claim follows from the well-ordering, the translation invariance and transitivity of $>$. ■

Theorem 35 *Let $\mathbf{F}_q$ be a finite field and $n \in \mathbb{N}$ a natural number. Then the family of polynomials*

$$(\tau_1^q - \tau_1, \tau_2^q - \tau_2, ..., \tau_n^q - \tau_n)$$

is a basis for the vanishing ideal

$$I(\mathbf{F}_q^n) \subseteq \mathbf{F}_q[\tau_1, ...\tau_n]$$

Proof. The proof of this well known result can be found after Lemma 3.1 of (Germundsson, 1991). ■

Theorem 36 *Let $\mathbf{F}_q$ be a finite field and $n \in \mathbb{N}$ a natural number. Then the family of polynomials*

$$(\tau_1^q - \tau_1, \tau_2^q - \tau_2, ..., \tau_n^q - \tau_n)$$

is a universal Gröbner basis for the vanishing ideal

$$I(\mathbf{F}_q^n) \subseteq \mathbf{F}_q[\tau_1, ...\tau_n]$$

Proof. From the inequalities 9 it follows in particular for all possible monomial orders

$$LM(\tau_i^q - \tau_i) = \tau_i^q \ \forall \ i \in \{1, ..., n\}$$

As a consequence, for the least common multiple (LCM) of $LM(\tau_j^q - \tau_j)$ and $LM(\tau_i^q - \tau_i)$, $i \neq j$ holds

$$LCM(LM(\tau_j^q - \tau_j), LM(\tau_i^q - \tau_i)) = LCM(\tau_j^q, \tau_i^q) = \tau_j^q \tau_i^q \ \forall \ i, j \in \{1, ..., n\} \text{ with } i \neq j$$

and for the S-polynomial of $\tau_j^q - \tau_j$ and $\tau_i^q - \tau_i$, $i \neq j$ we have

$$S(\tau_j^q - \tau_j, \tau_i^q - \tau_i) = \tau_i^q(\tau_j^q - \tau_j) - \tau_j^q(\tau_i^q - \tau_i) = \tau_j^q \tau_i - \tau_i^q \tau_j \ \forall \ i, j \in \{1, ..., n\} \text{ with } i \neq j$$

Now let's divide $S(\tau_j^q - \tau_j, \tau_i^q - \tau_i) = \tau_j^q \tau_i - \tau_i^q \tau_j$ by $(\tau_1^q - \tau_1, \tau_2^q - \tau_2, ..., \tau_n^q - \tau_n)$. Without loss of generality let

$$\tau_j^q \tau_i > \tau_i^q \tau_j$$

(which is equivalent to $LT(\tau_j^q \tau_i - \tau_i^q \tau_j) = \tau_j^q \tau_i$). Then, after the first division step, we get the remainder

$$-\tau_i^q \tau_j + \tau_i \tau_j$$

Now we know from the inequalities (9) after translation by τ_j

$$\tau_i^q \tau_j > \tau_i \tau_j \Rightarrow LT(-\tau_i^q \tau_j + \tau_i \tau_j) = -\tau_i^q \tau_j$$

so we can continue the division process and we get the remainder

$$-\tau_i^q \tau_j + \tau_i \tau_j - (-\tau_j)(\tau_i^q - \tau_i) = 0$$

By the theorem above

$$I(\mathbf{F}_q^n) = \langle \tau_1^q - \tau_1, \tau_2^q - \tau_2, ..., \tau_n^q - \tau_n \rangle$$

And so, according to Buchberger's S-pair criterion (see Theorem 6 of chapter 2, §6 in (Cox *et al.* , 1997)),

$$(\tau_1^q - \tau_1, \tau_2^q - \tau_2, ..., \tau_n^q - \tau_n)$$

is a universal Gröbner Basis for $I(\mathbf{F}_q^n)$. ■

References

Becker, T., & Weispfenning, V. 1993. *Gröbner bases*. Graduate Texts in Mathematics, vol. 141. New York: Springer-Verlag. A computational approach to commutative algebra, In cooperation with Heinz Kredel.

Buchberger, B. 1970. Ein algorithmisches Kriterium für die Lösbarkeit eines algebraischen Gleichungssystems. *Aequationes Math.*, **4**, 374–383.

Buchberger, B. 1976. A theoretical basis for the reduction of polynomials to canonical forms. *ACM SIGSAM Bull.*, **10**(3), 19–29.

Cox, D., Little, J., & O'Shea, D. 1997. *Ideals, varieties, and algorithms, An introduction to computational algebraic geometry and commutative algebra*. Second edn. Undergraduate Texts in Mathematics. New York: Springer-Verlag.

Germundsson, R. 1991 (Sep). *Basic results on ideals and varieties in finite fields*. Tech. rept. LiTH-ISY-I-1259. Linköping University, Linköping, Sweden.

Lauer, M. 1976. Canonical representatives for residue classes of a polynomial ideal. *Pages 339–345 of: SYMSAC '76: Proceedings of the third ACM symposium on Symbolic and algebraic computation*. New York, NY, USA: ACM Press.

Lidl, R., & Niederreiter, H. 1997. *Finite fields*. Second edn. Encyclopedia of Mathematics and its Applications, vol. 20. Cambridge: Cambridge University Press. With a foreword by P. M. Cohn.

Macaulay, F. S. 1994. *The algebraic theory of modular systems*. Cambridge Mathematical Library. Cambridge: Cambridge University Press. Revised reprint of the 1916 original, With an introduction by Paul Roberts.

Sauer, Thomas. 2001. Gröbner bases, H-bases and interpolation. *Trans. Amer. Math. Soc.*, **353**(6), 2293–2308 (electronic).

Scharlau, W. 1969. *Quadratic forms*. Queen's papers in pure and applied mathematics, vol. 22. Kingston, Ontario: Queen's University.
